# Systematic mapping review of the factors influencing physical activity and sedentary behaviour in ethnic minority groups in Europe: a DEDIPAC study

**DOI:** 10.1186/s12966-017-0554-3

**Published:** 2017-07-24

**Authors:** Lars Jørun Langøien, Laura Terragni, Gro Rugseth, Mary Nicolaou, Michelle Holdsworth, Karien Stronks, Nanna Lien, Gun Roos, on behalf of the DEDIPAC consortium

**Affiliations:** 1 Norwegian School of Sport Sciences, Oslo, Norway; 2 Department of Nursing and Health Promotion, Faculty of Health Sciences, Oslo and Akershus University College of Applied Sciences, Oslo, Norway; 3 Department of Public Health, Academic Medical Centre, University of Amsterdam, Amsterdam, the Netherlands; 4 Public Health Section, School of Health and Related Research, University of Sheffield, Sheffield, UK; 5 Department of Nutrition, University of Oslo, Oslo, Norway; 6 Consumption Research Norway – SIFO, Oslo and Akershus University College of Applied Sciences, Oslo, Norway

**Keywords:** Physical activity, Sedentary behaviour, Factors, Ethnic minority groups, Europe, Migrants, Immigrants

## Abstract

**Background:**

Physical activity and sedentary behaviour are associated with health and wellbeing. Studies indicate that ethnic minority groups are both less active and more sedentary than the majority population and that factors influencing these behaviours may differ. Mapping the factors influencing physical activity and sedentary behaviour among ethnic minority groups living in Europe can help to identify determinants of physical activity and sedentary behaviour, research gaps and guide future research.

**Methods:**

A systematic mapping review was conducted to map the factors associated with physical activity and sedentary behaviour among ethnic minority groups living in Europe (protocol PROSPERO ID = CRD42014014575). Six databases were searched for quantitative and qualitative research published between 1999 and 2014. In synthesizing the findings, all factors were sorted and structured into clusters following a data driven approach and concept mapping.

**Results:**

Sixty-three articles were identified out of 7794 returned by the systematic search. These included 41 quantitative and 22 qualitative studies. Of these 58 focused on physical activity, 5 on both physical activity and sedentary behaviour and none focused on sedentary behaviour. The factors associated with physical activity and sedentary behaviour were grouped into eight clusters. Social & cultural environment (*n* = 55) and Psychosocial (39) were the clusters containing most factors, followed by Physical environment & accessibility (33), Migration context (15), Institutional environment (14), Social & material resources (12), Health and health communication (12), Political environment (3). An important finding was that cultural and religious issues, in particular those related to gender issues, were recurring factors across the clusters.

**Conclusion:**

Physical activity and sedentary behaviour among ethnic minority groups living in Europe are influenced by a wide variety of factors, especially informed by qualitative studies. More comparative studies are needed as well as inclusion of a wider spectrum of the diverse ethnic minority groups resettled in different European countries. Few studies have investigated factors influencing sedentary behaviour. It is important in the future to address specific factors influencing physical activity and sedentary behaviour among different ethnic minority groups in order to plan and implement effective interventions.

**Electronic supplementary material:**

The online version of this article (doi:10.1186/s12966-017-0554-3) contains supplementary material, which is available to authorized users.

## Background

Low levels of physical activity (PA) and high levels of sedentary behaviour (SB) are associated with obesity and non-communicable diseases (NCDs), including type 2 diabetes and cardiovascular diseases (WHO, 2014). Enhancing the levels of PA and reducing the levels of SB can prevent and to some extent treat NCDs. Studies indicate that some ethnic minority groups are less active and more sedentary than the majority population and that factors influencing these behaviours may differ (e.g. [1–3]). In Europe, reviews have been undertaken on obesity and PA among a limited number of ethnic minority or migrant groups. These include reviews of PA in North African migrants [4] and South Asian women in different Western countries [5]. These reviews mainly focused on levels of PA, and indicated that information on barriers and facilitators is limited. However, to develop effective interventions that also reach ethnic minority groups information about factors influencing PA and SB is necessary. To our knowledge there are no systematic reviews on factors influencing PA and SB in ethnic minority groups across Europe. The ethnic minority groups in Europe are quite diverse in terms of size, country of origin and migration patterns, and there are substantial variations between countries [6]. In recent years the number of asylum seekers have increased and the three largest groups have been from Syria, Afghanistan and Iraq [7]. Refugees, asylum seekers and migrants seem to be at risk for worse health outcomes including NCDs [8]. As the migrant composition in Europe is changing and growing, gathering knowledge on factors influencing PA and SB of migrants and identifying gaps in the literature is crucial for assessing the needs of these populations and planning interventions.

The aim of this study was to systematically review the literature that has identified factors influencing PA and SB across the life-course among ethnic minority groups living in Europe, uncover gaps in the literature and suggest priorities for future research. This review is part of the work performed on ethnic minority groups in the DEDIPAC (Determinants of Diet and Physical Activity) Knowledge Hub [9]. DEDIPAC KH is a European research network aiming at understanding the ‘causes of the causes’ of diet, physical activity and sedentary behaviour. The evidence from this review also feeds into a DEDIPAC study of factors influencing diet and PA/SB behaviours in ethnic minority groups in Europe [10].

## Methods

We conducted a systematic mapping review to find both quantitative and qualitative published literature. According to Grant and Booth [11] mapping reviews are suited to map out the existing literature to identify tendencies and gaps in the research literature to commission future research. The review protocol was registered with PROSPERO (ID = CRD42014014575).

**Fig. 1:**
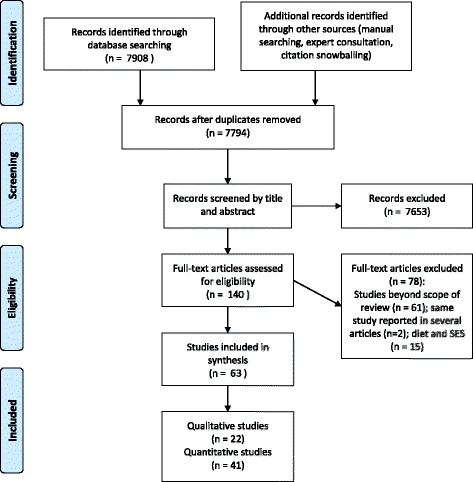
PRISMA flow chart: Selection process for articles

The stages of the data selection process are presented in Fig. 1. The search was conducted in the following electronic databases: MEDLINE, EMBASE (Ovid), Web of Science, Cochrane Library, CINAHL, Psychinfo (Ovid). The databases were searched from 1999 to 2014. This time period was chosen because we anticipated that factors identified before 1999 would be referred to in more recent literature. The citation follow–up technique was used to identify studies that had not been identified through the systematic search of the databases. Additionally, experts in the subject area, researchers working in the fields of physical activity and sedentary behaviour, ethnicity and health were contacted to identify relevant studies that might have been missed in the search of electronic databases.

Ethnic minority group is a concept used for very heterogeneous groups that may share minority status in their country of residence due to ethnicity, place of birth, language, religion, citizenship as well as other cultural differences. This definition may include groups from newly arrived immigrants to (minority) groups that have been part of a country’s history, for instance the Roma and Sami people. Within the DEDIPAC consortium we reached an agreement on what we meant by ‘Ethnic minority groups’, who were defined as immigrants/populations of immigrant background (not differentiating based on their migration status) from low and middle income countries, population groups from the former Eastern Bloc countries who migrate to other parts of Europe and minority indigenous populations in Europe [9]. We decided to focus on these ethnic minority groups because they are more likely to be of lower socioeconomic status.

### Inclusion and exclusion criteria

Observational and intervention studies using quantitative and/or qualitative methods examining physical activity and sedentary behaviour among ethnic minority groups in Europe were included. In addition, studies that identified an association between a factor (including correlates, predictors, moderators, determinants and mediators) and physical activity or sedentary behaviour of ethnic minority groups living in Europe were included. All age groups were included.

Studies that analysed physical activity or sedentary behaviour as a confounder in a relationship between ethnicity and disease, including studies that explored whether ethnicity is a factor influencing physical activity or sedentary behaviour without explanation were excluded because we were interested in studies that contained relevant information on factors. Studies only presenting descriptive information about physical activity or sedentary behaviour, or just describing levels of activity according to belonging to an ethnic minority group without providing any explanation were also excluded. As were non-human studies and laboratory based studies.

### Search strategy and study selection

A search strategy was constructed to identify studies with the use of free text word and MESH terms, which was modified for each database. The search strategy was guided by search terms within three concepts: physical activity and/or sedentary behaviour; ethnic minority group; and Europe (as the setting). The complete strategy is shown in Additional file 1. Retrieved literature was stored in an Endnote database and duplicate entries were deleted.

To ensure accuracy and consistency of data extraction two reviewers (LJL and LT) independently screened titles and abstracts of identified studies for relevance, according to the review inclusion criteria. Spot checks were conducted on a sample of screened sources to assess the extent of agreement between reviewers. All retrieved full texts papers were reviewed by two of the team of four independent researchers (GrR and LJL or LT and GR).

### Data extraction and synthesis

Data from papers included in the full text review phase were extracted by four independent researchers (LJL, LT, GrR and GR). An extraction tool was developed to collate data which was used to identify general information and study characteristics (author, year of publication, country, and study design, sample size), population characteristics (gender, age, country of origin, years since migration, acculturation, education, migration history, and other relevant demographics).

Quality assessment of quantitative and qualitative studies was undertaken using established quality assessment criteria for evaluating primary research papers [12]. The quality of articles included after the full text review were assessed by two of the team of four independent researchers involved in data extraction. The quality assessment scores given to the included studies are included in Tables 1 and 2.

**Table 1:** Study characteristics of quantitative studies (*N* = 41)

Author	Study design	Study population	Sample characteristics	Number of participants	Setting, country	Recruitment	Physical activity (PA), Sedentary Behaviours (SB)	Measurement	Quality score
Andersen et al., 2011 [65]	RCT	Pakistani immigrant men	25–60 years	150	Oslo, Norway	Mosques and Muslim festivals	PA	Accelerometer, treadmill, questionnaire	20/22
Andersen et al., 2013 [50]	RCT	Pakistani immigrant men	25–60 years	126	Oslo, Norway	Mosques and Muslim festivals	PA	Accelerometer	21/24
Arvidson et al., 2014 [55]	Cross sectional	Iraqis and Swedes	30–75 years	599 Iraqis 553 Swedes	Malmö, Sweden	Randomly selected from the census register	PA	Self-report, accelerometer	20/22
Babakus et al., 2012 [5]	Review	South Asian women	18 years and older	26 quantitative 12 qualitative studies	UK, US, Canada, Australia, Norway, India, New Zealand, Guadeloupe	Various	PA, SB	Various	18/18
Besharat Pour et al., 2014 [77]	Cross sectional	Children of immigrants and Swedish parents	8 years	2589 21.7% one or two immigrant parents	Stockholm, Sweden	Prospective birth cohort study BAMSE	PA	Parental questionnaire	22/22
Dawson et al., 2005 [21]	Cross sectional	Born in Sweden, Western Europe, Finland, Southern Europe, Eastern Europe, other countries	20–74 years	7172 men (699 immigrants) 7313 women (799 immigrants)	Sweden	General population	PA	Questionnaire	21/22
De Munter et al., 2012 [67]	Cross sectional	South Asian-Surinamese, African-Surinamese and European-Dutch	35–60 years	370 South Asian-Surinamese, 689 African-Surinamese, 567 European-Dutch	Amsterdam, The Netherlands	Random sample from the population register	Leisure-time PA and active commuting	Interview questionnaire, physical examination	21/22
De Munter et al., 2013 [73]	Cross sectional	European, Indian, African-Caribbean	36–60 years	European (English = 14,723, Dutch = 567), Indian (E = 1264, D = 370), African –Caribbean (E = 1112, D = 689)	Amsterdam, The Netherlands and England	Random sample from the population register	Leisure-time PA	Questionnaire	19/22
Drenowatz, et al., 2013 [56]	Cross sectional	Parent born outside Germany or foreign language spoken during the child’s first years	8 years	995	Southern Germany	Representative sample of children from 32 schools participating in an intervention	PA, physical fitness, sport	General motor abilities test for children. Parental questionnaire for participation in organized sports	21/22
Edwardson et al., 2014 [51]	Cross sectional	General population, multi-ethnic sample of students	11–14 years	588 girls 578 boys	The East Midlands, England	Multi-ethnic representative sample of students from 5 schools. Schools recruited based on SES	PA, activity-related social support	Questionnaire	21/22
Garduño-Diaz et al., 2013 [22]	Cross sectional	African-Caribbean, South Asian, Caucasian groups in the UK	20 years and older	210	Leeds, England	Random sample - three area codes in Leeds	PA	Questionnaire	20/22
Gele et al., 2013 [62]	Cross sectional	Somali immigrants	25 years and older	115 women 93 men	Oslo, Norway	Non-random snowball sampling	PA	Questionnaire	21/22
Gualdi-Russo et al., 2014 [4]	Review	North African children living in their home countries or as immigrants in Europe	0–21 years	Various	European countries	Various	PA	Various	17/20
Hansen et al., 2008 [72]	Cross sectional	Non-Western immigrants with Danish citizenship compared with citizens with Danish background	25–64 years	135 (Turkey, Bosnia, Sri Lanka, Iran, Lebanon, Vietnam, Pakistan, Palestine, India, Croatia, Egypt)	Denmark	Based on National Health Interview Survey 2005	PA, SB		20/22
Hayes et al., 2002 [61]	Cross sectional	European, Indian, Pakistani and Bangladeshi residents	General population, 25–75 years	825 Europeans, 684 Indian, Pakistani or Bangladeshi	Newcastle upon Tyne, England	Random selection	PA, SB	Questionnaire	20/22
Hermansen et al., 2002 [66]	Cross sectional	People of Norse and Sami origin (indigenous)	All residents, 40–62 years	860 women 866 men of Sami origin 3948 women 4105 men of Norse origin	Finnmark county, Norway	Invited by personal letter with questionnaire	PA, leisure-time and work	Questionnaire	20/22
Hornby-Turner et al., 2014 [24]	Cross sectional	British-Pakistani and White British girls	9–11 years	70 White British 75 British Pakistani (+ parents of 19 girls)	North East England	Approached 8 primary schools. Parents of participating children asked by letter to participate in interview	PA, SB	Questionnaire, PA interview, Accelerometer	19/22
Hosper et al., 2007 [59]	Cross sectional	First and second generation Turkish young people	15–30 years	249 women 236 men	Amsterdam, The Netherlands	Random sample drawn from Amsterdam population registry	Leisure-time PA	Structured interview, Questionnaire	20/22
Hosper et al., 2008 [53]	Cross sectional	Turkish and Moroccan women	15–30 years	258 Turkish 170 Moroccan women	Amsterdam, The Netherlands	Random sample drawn from the Amsterdam population registry	PA, sport, leisure-time PA	Questionnaire	21/22
Jönsson et al., 2012 [71]	Cross sectional	Women living in Sweden (born in Finland, Chile and Iraq)	18–65 years	1651 women	Stockholm and Botkyrka, Sweden	Random sample drawn from the population registers	Leisure-time PA	Postal questionnaire	20/22
Khunti et al., 2007 [29]	Cross sectional	5 schools	11–15 years	3601	Leicester, England	Representative sample through schools	PA	Questionnaire	20/22
Koca et al., 2014 [30]	Cross sectional	Turkish migrants in Germany and England	Adults, (first, second, third generation)	Germany: 140 women 126 men England: 125 women 125 men	Frankfurt, Germany, London, UK	Convenience sample. Recruited from migrant organizations	PA level	Questionnaire, face-to-face interview	20/22
Kumanyika et al., 2012 [57]	Review	African descent populations in English-speaking countries, other ethnic minority populations			U.S, New Zealand, Canada and Europe		PA resources, facilities, opportuni-ties	Interdisciplinary group discussions	15/18
Kumar et al., 2006 [69]	Cross sectional	Ethnic minority groups (born in Turkey, Iran, Pakistan, Sri Lanka, Vietnam)	Born in 1942–1970	1320 women 1679 men	Oslo, Norway	2001 population registry - invited to participate	Leisure-time PA	Health questionnaire, clinical screening	20/22
Lecerof et al., 2011 [63]	Cross sectional	Recently settled Iraqi migrants in Sweden	18–64 years	306 women 273 men	Sweden	All adults born in Iraq who were registered in 8 counties (Dec 2007 – Feb 2008)	PA	Postal questionnaire	19/20
Lindström et al., 2001 [44]	Cross sectional	Malmö general population (83 different countries of origin)	20–80 years	1916 women 1872 men	Malmö, Sweden	Random sample. General population in 1994 (born in 1913, 1923, 1933, 1943, 1953, 1963, 1968, 1973)	Leisure-time PA	Postal questionnaire	20/20
Magnusson et al., 2005 [64]	Cross sectional	Children in grades 5 and 6	11–12 years	108	Community (6700 inhabitants) near Gothenburg, Sweden	All children in grades 5 and 6 at a Swedish school	PA, exercise	Questionnaire, individual interview	19/22
Me’jean et al., 2009 [74]	Retrospective cohort study	Tunisian migrant men	Mean 50 years	150 men	South France	Quota sampling based on age and place of residence	PA	Interview, questionnaire	22/22
Molaodi et al., 2012 [80]	Environment - modelling	White British, Black Africans, Black Caribbean, Indians, Pakistanis, Bangladeshis, Chinese and Irish	Deprived areas		UK		Physical activity facilities	Lists from Sport England	21/22
Nielsen et al., 2013 [60]	Cross sectional	Children with other ethnic background than Danish	Pre-school age and same children in third grade	Preschool 67 other ethnic background of 594; third grade 58 of 518	Taarnby and Ballerup, Copenhagen	Children from 18 schools in Taarnby and Ballerup were invited	Habitual PA	Accelerometer, parental questionnaire	22/22
Nilsson et al., 2011 [76]	Cohort study	Elderly Sami	60 years and older	9 women 11 men 81 reindeer-herding and 226 non-reindeer-herding Sami	Southern Lapland. Västerbotten county	Suggested by local associations, invited by posted letter. Västerbotten intervention project cohorts 40, 50, 60 years	PA	Semi-structured interviews, questionnaire	22/22
Owen et al., 2009 [33]	Cross sectional	British children of South Asian, black African-Caribbean and white European origin	9–10 years	*N* = 1841 (562 white Europeans, 494 South Asians, 607 African-Caribbeans, 408 other ethnic groups)	London, Birmingham, Leicester, UK	Random samples (all state primary schools in London, Birmingham and Leicester with 15–50% pupils of White European origin)	PA	Accelerometer	22/22
Pudaric et al., 2000 [35]	Cross sectional	Elderly foreign-born persons	55–74 years	159 women 94 men	Sweden	Random sample drawn from Swedish Population Registry	PA	Face-to-face interview	22/22
Reijneveld et al., 2003 [36]	RCT	Turkish immigrants	45 years and older	41 women 51 men	The Netherlands - 6 cities	Welfare services	PA	Self-report	19/24
Sagatun et al., 2008 [37]	Longitudinal	10th grade in Oslo in 1999–2001	15–16 years (follow up 18–19 years)	1377 girls 1112 boys	Oslo, Norway	All students in grade 10	Leisure-time PA	Questionnaire	21/22
Saris et al., 2013 [68]	Cross sectional	Adults in deprived neighbourhoods (39.7% migrants - mainly non-Western)	18 years and older	337 women 285 men	The Netherlands	Randomly selected adults in 20 most deprived neighbourhoods	Active transport	Questionnaire, interview	22/22
Södergren et al., 2010 [46]	Cross sectional	Women born in Sweden, Finland, Chile and Iraq	18–65 years	2649 women	Stockholm, Sweden	Random sample from population registers in 2 municipalities of Stockholm	PA	Postal questionnaire	20/22
van Rossem et al., 2012 [75]	Cohort	Children at age 3 enrolled in a birth cohort		2351 girls 2337 boys	Rotterdam, The Netherlands	All pregnant mothers with expected delivery April 2002–January 2006	PA, SB	Parental questionnaire	19/22
Walseth et al., 2004 [42]	Review	Minority women					Sport		11?
Williams et al., 2010 [70]	Cross sectional	South Asian and White	18–55 years	5421 South Asian 8974 White	UK		PA Leisure activities/sports, domestic activities, walking		20/20
Yates et al., 2010 [54]	Cross sectional	White European and South Asians	White European: 40–75 years South Asians: 25–75 years	White European: 2277 women 2033 men South Asians: 560 women 604 men	Leicester, UK	Primary care	PA	Questionnaire	21/22

Study design: *RCT* Random Control Trial

**Table 2:** Study characteristics of qualitative studies (*N* = 22)

Author	Study design	Study population	Sample characteristics	Number of participants	Setting, country	Recruitment	Physical activity (PA), Sedentary Behaviours (SB)	Measurement	Quality score
Benn et al., 2013 [18]	Case studies	Muslim girls and stakeholders	England: 8 state schools Denmark: One class, 16–17 years	England: 109 female students 19 teachers 32 parents Denmark: 42 female and male students	England and Denmark	Representative sample	PA, Physical education	England: Qualitative survey, focus groups, semi-structured interview Denmark: survey, video observation of sport/PE lessons, interview	16/20
Beune et al., 2010 [47]	Qualitative inductive	Ghanaians, African-Surinamese, White Dutch		26 women 20 men (19 Ghanaian, 19 Surinamese, 16 White Dutch)	Amsterdam, The Netherlands	Purposive sampling through health-care centres	PA		19/20
Dagkas et al., 2006 [19]	Interpretive study	Greek Turkish girls and British Asian women, living in predominantly non-Muslim countries	Greek: 13–15 years British: 18–21 years	Greek: 24 girls at school British: 20 women at university	Greece and Great Britain	British group was participating in a larger life history project	PA, Physical education, sport	Semi-structured interview	16/20
Dagkas et al., 2011 [20]	Case studies	Muslim girls	5–16 year	109 girls 19 teachers 32 parents. Additional focus groups - 36 girls	West Midlands, England	Representative sample of schools	PA, Physical education, school sport	Focus groups	19/20
Hendriks et al., 2012 [23]	Theoretical framework	Surinamese Immigrants of Indian (Hindustani) Descent	29–83 years, lived in The Netherlands for 25–39 years - feeling 100% Hindu	24 women 3 men	The Netherlands	Through community houses and yoga class. Snowball technique	PA	Semi-structured interview, focus groups	19/20
Horne et al., 2010 [58]	Ethnographic approach	Community dwelling White and South Asians	60–70 years	Focus groups: 87 Interviews: 40	The North West England	From a period of fieldwork observation in leisure groups and social centres	PA, exercise	Focus groups, in-depth interview	16/20
Horne e.t al., 2012a [25]	Ethnographic approach	South Asians	60–70 years	Focus groups: 29 Interviews: 17	UK	From a period of fieldwork observation in leisure groups and social settings	PA	Focus groups, in-depth interview	18/20
Horne et al., 2012b [52]	Systematic review	South Asian older adults		10 studies	UK and Canada		PA		20/20
Horne et al., 2013 [26]	Exploratory qualitative approach	Community dwelling White and South Asians	60–70 years		The North West of England	Purposive sampling was used to recruit participants with different experiences of PA participation	PA, exercise	Focus groups and in-depth interview	16/20
Johnson, 2000 [27]	Survey, review of qualitative studies	South Asians	16–74 years	22 focus groups (14 with South Asians)	UK		PA	Focus groups	14/20
Kay, 2006 [28]	Interviews with women and their families	Young Muslim women	13–18 years and their families	6 families. 7 women (Bangladeshi, black African, Arab)	Midland town, UK		PA, sport	Interview	18/20
Lawton et al., 2006 [31]	Qualitative in-depth interviews	Pakistani and Indian patients in Scotland diagnosed with Type 2 diabetes	Over 18 years, diagnosed with Type 2 diabetes	Pakistani: 12 women 11 men Indian: 5 women 4 men	Edinburgh, Scotland	Both clinical and local community recruitment. Purposively sampled. Snowball sampling	PA	In-depth interview	15/17
Lucas et al., 2013 [40]	Review, meta-ethnographic approach	UK South Asian populations	Adults	10 qualitative studies	UK		PA, exercise		19/20
Nicolaou et al., 2012 [32]	Focus groups	Moroccan women	Women	Amsterdam: 4 focus groups (22 women) Morocco: 4 focus groups (29 women)	Amsterdam, The Netherlands and Morocco	Amsterdam (Mother-child centre, women’s centre) and Morocco (Al Hoceima town, small village and medium sized village)	PA	Focus groups	20/20
Pallan et al., 2012 [34]	Focus groups with stakeholders			9 focus groups with 68 local community stakeholders	UK	Stakeholders from 8 school communities with predominantly South Asian pupils	PA	Focus groups	17/20
Pallan et al., 2013 [41]	Development of intervention	UK South Asian primary school-aged children			Birmingham, UK		PA	Focus groups, literature review, expert group, review of local resources	19/20
Pollard et al., 2012 [48]	Largely qualitative	British Pakistani women, Muslim		22 women	Newcastle upon Tyne, UK	Information event at leisure centre, snowball sampling	PA	Interview, accelerometer, 24 h recall	16/19
Rawlins et al., 2013 [45]	Focus groups	Black Caribbean, Black African, Indian, Pakistani, Bangladeshi and White British children and their parents	Schools: 11–12 years, 10–11 years; Places of worship: 8–13 years and parents	39 female 31 male students 34 mothers 9 fathers	London boroughs (Brent, Croydon, Ealing, Hackney, Hillingdon, Lambeth)/UK	Schools or places of worship	Healthy lifestyles	Focus groups, interview	18/18
Sriskantharajah et al., 2007 [49]	Explorative qualitative	South Asian women (Indian, Pakistani, Bangladeshi, East African Asian, Sri Lankan)	26–70 years, CHD and diabetes type 2	15 women	UK	Purposive sampling: 3 general practices	PA, exercise	Semi-structured interview	16/17??
Steinbach et al., 2011 [38]	Qualitative in-depth interviews, fieldwork	workplaces with a mixed workforce (ethnicity, income, age)		78	London, UK	Purposive sampling	Cycling for transport	Qualitative in-depth interview, fieldwork	14/16
Södergren et al., 2008 [39]	Explorative qualitative	Immigrant women from Chile, Iraq and Turkey	26–65 years	63 women	Stockholm, Sweden	Multi-recruitment strategy	PA, exercise	Focus groups	18/18
Walseth, 2006 [43]	Life-history	Muslim women with immigrant background (Pakistan, Turkey, Morocco, Iran, Syria, Somalia, Gambia, Macedonia, Kosovo)	16–25 years	21 women	Norway	Sampled through their former status as pupils at one elementary school in Oslo and through sport clubs	PA, Sport	Life-history interviews	19/20

Common methodologies for identifying and grouping factors were used as for a similar review on dietary determinants of ethnic minority groups [13]. First, all factors from the included studies were identified. Then these factors were sorted into clusters according to how they were seen to relate to each other, following a data driven approach [14]. The clustering was part of a larger concept mapping process with the aim of developing a system-based framework of factors influencing dietary behaviour and physical activity/sedentary behaviour in the general European population [15–17] and in ethnic minority groups living in Europe (manuscript in preparation: [10]). For PA/SB the factors were grouped into eight clusters; Health & health communication, Political environment, Social & cultural environment, Psychosocial, Institutional environment, Physical environment & accessibility, Social & material resources and Migration context.

## Results

### Description of included studies

We identified 63 articles on PA and SB (41 based on quantitative studies; 22 on qualitative) among ethnic minority groups in Europe (Fig. 1). It should be noted that we in some cases have chosen to include more than one article from the same study, or research populations, when the articles address different issues, or mention different factors of PA or SB. The large majority of the studies were on PA (*n* = 59), while five focused on both PA and SB. No paper focused solely on SB. About half of the studies (*n* = 33) were conducted in the Western parts of Europe, most in the UK. A large number of the studies were conducted in the Nordic countries (*n* = 19). A few studies were comparative (*n* = 7), comparing two European countries or at least one European country with countries on other continents. Additional file 2 summarises description of the included studies.

We categorised the populations studied by country of origin, region, ethnicity or religion [see Additional file 2 and a more detailed division can be seen in Tables 1 and 2]. Most of the studies focused on more than one population. The main bulk of the studies focused on South Asian (*n* = 36), from India, Pakistan, or Bangladesh. These populations are especially important minority groups in the UK. Twelve studies focused on populations of Middle Eastern heritage, while nine studied African or Black African populations. Four studies focused on people of North African descent. The remaining populations studied were: South Americans (*n* = 3), East Africa, ns (*n* = 1), West Africans (*n* = 1). A few studies did not specify where the minority populations descended from (*n* = 8), but used such terms as ‘other origin’, ‘migrants’, or ‘Muslims’. 27 of the studies were either comparing ethnic minority groups and the majority populations, or studies of the general populations indicating ethnic background. Two studies were on indigenous population in the Nordic countries (Sami: *n* = 2)).

Most of the 63 studies included both men and women (*n* = 45). Of the remaining studies 14 focused on women and 4 on men. Adults were the most studied group (*n* = 35). The second largest group was children (*n* = 13), followed by adolescents (*n* = 6), young older adults (*n* = 4), and older adults (*n* = 2). Three studies focused on the general population, and age was not specified. The number of participants ranged from 92 to 14,485 (of which 1498 were immigrants) in the quantitative studies and 15–202 (a few focus group studies had high numbers of participants) in the qualitative studies.

### Factors influencing physical activity/sedentary behaviour

We identified 183 distinct factors (Table 3) influencing PA and SB among ethnic minority groups in Europe. Of the identified factors, 60 were identified in qualitative studies and 54 were identified through quantitative studies only. The remaining factors were identified in both qualitative and quantitative studies, though many were predominantly identified through qualitative studies. The factors were often described as facilitators or barriers to PA and SB. Most factors were assigned to the Social & cultural environment cluster (*n* = 55 factors), followed by Psychosocial (*n* = 39), Physical environment & accessibility (*n* = 33), Migration context (*n* = 15), Institutional environment (*n* = 14), Social & material resources (*n* = 12), Health & health communication (*n* = 12), and finally Political environment (*n* = 3). The factors in the smallest cluster “Political environment” were identified only in qualitative studies. Qualitative studies were also strongly represented in most of the other clusters, but quantitative studies dominated the clusters “Migration context” and “Social & material resources”.

**Table 3:** Eight systems and 165 factors influencing PA and SB in ethnic minority populations (Study populations and references in bold are quantitative)

Health & health communication	Study population [ref]	Political environment	Study population [ref]	Social & cultural environment	Study population [ref]	Psychosocial	Study population [ref]	Institutional environment	Study population [ref]	Physical environment & opportunity	Study population [ref]	Social & material resources	Study population [ref]	Migration context	Study population [ref]
Health conditions	*n* = 7 Surinamese, & Indian [23] South Asian [25–27] Pakistani & Indian [31] Asian [40] Multiethnic [47]	Local Political orientation	*n* = 3 Muslim [18–20]	Gender	*n* = 23 **South Asian [5]** Muslim [18–20, 28] General [21] African, Asian [22] Surinamese & Indian [23] Pakistani [24] South Asian [25–27, 34] Diverse [29, 35–39] Turkish [30] Pakistani & Indian [31] Moroccan [32] South Asian & Caribbean [33]	Knowledge of PA and Health	*n* = 16 **North African [4] South Asian [5]** Muslim [19, 20] Surinamese & Indian [23] South Asian [26, 40] Diverse [45] **Pakistani [50]** Turkish & Moroccan [52] **General[54] Middle East [55] South Asian [61] Asian [62] General[63] Immigrants [64]**	Demands of curriculum	*n* = 3 **Muslim [19]** Diverse [29] **Diverse [45]**	Sports facilities available	*n* = 11 **South Asian [5]** South Asian [18, 26, 27, 34] **Diverse [29]** Pakistani & Indian [31] Diverse [45] Multiethnic [47] **Pakistani [50] Multiethnic [67]**	Practicalities/responsibilities	*n* = 10 Pakistani [24] South Asian [26, 27, 40, 52] Muslim [28] Pakistani & Indian [31] **Minority [42]** Mixed [45] **Sami [76]**	Language	*n* = 9 South Asian [25–27, 40, 49, 52] **South Asian** [**54] Mixed [71, 72]**
Physical health	*n* = 4 South Asian [26] Pakistani & Indian [31] South Asian [49] **Pakistani [65]**	Health care system adaptation	*n* = 2 Multiethnic [47] South Asian [58]	Religious requirements	*n* = 9 Muslim [20] South Asian [26, 34, 40, 41] Pakistani, Indian, [31] General [37] [40, 41] **Diverse [42]** Diverse [43]	Self-image	*n* = 11 Muslim [20] Surinamese & Indian [23] South Asian [26, 49, 52, 58] Asian [27] Multiethnic [47] **Pakistani [50, 65] Turkish & Moroccan [53]**	Head teacher’s attitude and resources	*n* = 3 **Muslim [18–20]**	Lack of appropriate activities	*n* = 10 **South Asian [5, 61]** Muslim [18] South Asian [26, 27, 40] immigrant [39] **Minority [42] African [62] Pakistani [65]**	Time constraints	*n* = 7 **South Asian [5]** South Asian [27, 40] **Mixed [29]** Pakistani & Indian [31] Immigrant [39] **Pakistani [50]**	Acculturation	*n* = 6 **Mixed [21, 72] Turkish [30] Minority [42] Turkish & Moroccan [53] Turkish [59]**
Religious fasting	*n* = 3 Muslim [19] South Asian [26, 27]	National political orientation	*n* = 1 Muslim [18]	Religion and culture	*n* = 8 **South Asian [5]** Muslim [18, 20] South Asian [26] Minority [43] **General [44]** Diverse [45] Mixed **[46]**	Knowledge of PA	*n* = 9 Surinamese & Indian [23] South Asian [26, 40, 58] Pakistani & Indian [31] **Pakistani [50,65] Middle East [55] African [62]**	Priorities in school	*n* = 2 Diverse [29] **South Asian [41]**	Lack of culturally sensitive facilities	*n* = 7 Muslim [18, 19] South Asian [26, 27, 39, 52] Pakistani [31]	Occupation	*n* = 5 Pakistani & Indian [31] Immigrant [39] Multiethnic [47] **Multiethnic [67] Mixed [72]**	Country of birth	*n* = 5 **Mixed [21,44, 46, 71, 75]**
Healthcare support	*n* = 3 South Asian [26, 49, 58]			Commitments within family	*n* = 8 Muslim [20, 48] **Pakistani [24]** South Asian [27, 40] Pakistani & Indian [31] Immigrant [39] Multiethnic [47]	Dangers of environments and strangers	*n* = 7 **South Asian [5] Pakistani [24]** South Asian [26, 27, 34] Pakistani & Indian [31] [34] Mixed [45]	Exam pressure	*n* = 2 Muslim [20] **Diverse[29**]	Expenses	*n* = 6 Surinamese & Indian [23] South Asian [27, 34, 41] Diverse [45] **South Asian [61]**	Education	*n* = 4 **Turkish [30]** Immigrant [39] **Iraqi [63] Multiethnic [67]**	Racial harassment	*n* = 4 South Asian [27, 40] **Minority [42] South Asian [54]**
Mental health	*n* = 2 **South Asian [70] Pakistani [65]** ,			Modesty	*n* = 8 **South Asian [5]** Surinamese & Indian [23] South Asian [26, 27, 40, 49] Pakistani & Indian [31] Immigrant [38]	Ideas of ideal body	*n* = 6 South Asian [40, 41] Multiethnic [47] **South Asian [54,61] African [62]**	Occupational PA	*n* = 2 South Asian [27] **Turkish [59]**	Religious dress requirements	*n* = 6 Muslim [18, 20] African- Caribbean, Asian & South Asian [22] South Asian [27, 40] Immigrant [38]	Financial limitations	*n* = 4 **South Asian [5] General[21**] Immigrant [39] **Multiethnic [67]**	Time since migration	*n* = 4 Mixed **[21, 72] South Asian & Surinamese [73] Tunisian [74]**
Pain	*n* = 2 South Asian [26, 49]			Women as caregiver/mother	*n* = 7 **South Asian [5]** South Asian [26, 27, 40] **Minority [42] Mixed [46**] Muslim [48]	Confidence level	*n* = 6 South Asian [26, 49, 58] **Pakistani [50,65] Turkish & Moroccan [53]**	Traditional livelihood	*n* = 2 South Asian [27] **Sami [66]**	Transportation and infrastructure	*n* = 5 South Asian [26, 27] Moroccan [32] Multiethnic [47] **Mixed [68]**	Lack of parental time	*n* = 3 **Pakistani [24]** South Asian [34, 41]	Lack of knowledge of host culture	*n* = 3 **General [21] South Asian [54] Mixed [64]**
Primary health care	*n* = 2 Multiethnic [47] South Asian [58]			Ideals of behaviour	*n* = 7 Muslim [18, 28] Pakistani [24] South Asian [27, 34] Diverse [29] Immigrant [39]	Prevent disease	*n* = 6 **South Asian [5]** Surinamese & Indian [23] South Asian [25, 58] Minority [43] Pakistani [65]	Emphasis on competitive sports	*n* = 1 South Asian [34]	Climate	*n* = 5 Surinamese & Indian [23] South Asian [27] Pakistani & Indian [31] Moroccan [32] **Mixed [69]**	Taking time from family and gender role activities	*n* = 3 **South Asian [5]** African-Caribbean & South Asian [22] **Pakistani & Indian [31]**	Discrimination	*n* = 1 **Mixed [46]**
Lack of follow-up	*n* = 1 South Asian [58]			Family	*n* = 7 Muslim [18, 28] Surinamese & Indian [23] South Asian [26] **Pakistani [50] Multi-ethnic [51]** Turkish & Moroccan [52]	Karma/fatalism	*n* = 5 **South Asian [5]** Surinamese & Indian [23] Pakistani [31] South Asian [40, 52]	Lack of PA in school	*n* = 1 South Asian [34]	Unattractive neighbourhood	*n* = 4 Mixed [45] Multiethnic [47] Turkish [59] **Mixed [68]**	Income	*n* = 3 **Migrants [35]** Multiethic [47] **Mixed [77]**	Immigration history	*n* = 1 **Mixed [37]**
Poor physical fitness	*n* = 1 **Pakistani [65]**			Shame	*n* = 7 **South Asian [5]** Surinamese & Indian [23] South Asian [26, 27, 40] Pakistani & Indian [31] Immigrant [38]	Experience of PA/PE	*n* = 5 Immigrant [39] Multiethnic [47] **Middle East [55]** South Asian [58] **Mixed [60]**	Lack of extra-curricular staff	*n* = 1 **Diverse [29]**	Lack of gym instructor support	*n* = 4 Surinamese & Indian [23] South Asian [26, 52, 58]	Parental employment	*n* = 2 **Pakistani [24] Mixed [75]**	Stereotypes of children’s interests	*n* = 1 South Asian [34]
Stress	*n* = 1 **Pakistani [65]**			Cultural requirements	*n* = 6 Muslim [20] **Pakistani [24] Diverse[29]** South Asian [34] **Turkish & Moroccan [53] General [54]**	Goal setting	*n* = 5 Surinamese & Indian [23] South Asian [27, 40] **Minority [42] Pakistani [50]**	Limited school resources	*n* = 1 **Diverse [29]**	Living in urban area	*n* = 3 **Mixed [37] South Asian [40,70]**	social position	*n* = 2 Multiethnic [47] Pakistani & Indian [31]	Social ties with home country	*n* = 1 **Tunisian [74]**
Depression	*n* = 1 **Pakistani [65]**			Social acceptance of PA	*n* = 6 **South Asian [5] African, Asian [22]** South Asian [40] **General [54, 56] Middle East [55]**	Motivation	*n* = 5 South Asian [26, 40, 58] Immigrant [39] **Pakistani [65]**	PE choices unappealing	*n* = 1 **Diverse [29]**	Lack of women only facilities	*n* = 3 Muslim [18] **Mixed [37]** Immigrant [39]	Marital staus	*n* = 1 **Turkish [30]**	Generation	*n* = 1 **Minorities [57]**
Bad health	*n* = 1 **Pakistani [65]**			Social environment	*n* = 5 South Asian [27, 49] Multiethnic [47] Turkish & Moroccan [52] **Minorities [57]**	Notions of leisure-time PA	*n* = 4 South Asian [27] Pakistani & Indian [31] Moroccan [32] **Sami [66]**	Lack of separation work-leisure	*n* = 1 **Sami [66]**	Home environment	*n* = 3 Moroccan [32] **Immigrant** [**36**] Multiethnic [47]	Parental education level	*n* = 1 **Mixed [77]**	Host country	*n* = 1 **Turkish [30]**
				Social support	*n* = 5 South Asian [25, 26, 58] Immigrant [39] **Turkish & Moroccan [53**]	Lack of enjoyment of PA	*n* = 4 Surinamese & Indian [23] Pakistani & Indian [31] Diverse [45] Muslim [48]	PE’s difference from other subjects	*n* = 1 Muslim [19]	Too much traffic	*n* = 2 **Turkish [59]** Mixed [45]			Age at time of migration	*n* = 1 **Mixed [71]**
				Structural constraints in family	*n* = 5 **Pakistani [24]** Pakistani & Indian [31] Immigrant [39] **Turkish [59] Mixed [60]**	Health beliefs about PA	*n* = 3 Surinamese & Indian [23] Pakistani & Indian [31] **General[54]**	Negotiating participation	*n* = 1 Muslim [18]	Logistics of activities	*n* = 2 South Asian [26] Immigrant [39]			Migration	*n* = 1 South Asian [52]
				Concepts of aging/generation	*n* = 5 South Asian [26, 27] **Immigrant [36]** Turkish & Moroccan [52] **South Asian [61]**	Attitudes	*n* = 3 **Immigrants [56] South Asian [61] Pakistani [65]**			Short-term activities	*n* = 2 South Asian [26] Pakistani & Indian [31]			Immigrant parent	*n* = 1 **Mixed [77]**
				Parental attitudes to PA	*n* = 4 Muslim [18, 20, 28] **Minorities [60]**	Not the sporty type	*n* = 3 South Asian [27] Multiethnic [47] **Pakistani [65]**			Bad weather	*n* = 2 **South Asian [5] Pakistani [24]** ,				
				Lack of ‘exercise culture’	*n* = 4 **Diverse [29]** Pakistani & Indian [31] Turkish & Moroccan [52] **Middle East [55]**	PA as part of everyday life	*n* = 2 South Asian [25, 34]			Financial incentives	*n* = 2 Multiethnic [47]				
				Women not to be alone outside	*n* = 3 Muslim [28] Pakistani & Indian [31] **General[54]**	Interest in PA	*n* = 2 **Mixed [29]** Mixed [45]			Lack of information	*n* = 2 South Asian [52] **Mixed [77]**				
				Purposeful PA selfish	*n* = 3 **South Asian [5]** South Asian [49] **General[54]**	Perceived dis−/advantages of PA	*n* = 2 South Asian [49] **Turkish & Moroccan [53]**			Lack of open space	*n* = 2 **South Asian [5]** South Asian [34]				
				Age	*n* = 3 Muslim [20] **Turkish [30]** South Asian [58]	Self-efficacy	*n* = 2 **Pakistani [50] Turkish & Moroccan [53]**			Attractive environment	*n* = 1 **Mixed [68]**				
				Religious festivals	*n* = 3 Muslim [19] South Asian [26, 27]	Lack of knowledge of area	*n* = 2 Pakistani & Indian [31] South Asian [52]			Media portrayal of unsafe environment	*n* = 1 South Asian [34]				
				Religious prayer	*n* = 3 Muslim [19] South Asian [26, 27]	Perceived restrictions	*n* = 2 South Asian [49, 58]			Access to play area	*n* = 1 **Mixed [60]**				
				Peer group	*n* = 3 South Asian [26] Mixed [47] Turkish & Moroccan [52]	Views on age, lifestyle and health	*n* = 2 South Asian [49, 58]			Home appliances limit PA	*n* = 1 Moroccan [32]				
				Few active friends or family	*n* = 3 South Asian [27] **Minority [42] Pakistani [50]**	Fear of racism	*n* = 2 **South Asian [54,61]**			Lack of green space	*n* = 1 **Mixed [29]**				
				PA role models	*n* = 3 Muslim [20] Surinamese & Indian [23] **Pakistani [50]**	Preference of PA	*n* = 2 Multiethnic [47] South Asian [52]			Lack of safe storage in school for bikes/kit	*n* = 1 **Mixed [29]**				
				PA irrelevant to disease	*n* = 2 Pakistani & Indian [31] **General [54]**	Not gaining weight	*n* = 2 South Asian [49] **Pakistani [65]**			Few sidewalks	*n* = 1 **Turkish [59]**				
				Ethnic group	*n* = 2 **South Asian & Caribbean [33]** Minority [43]	Want to be fit	*n* = 2 South Asian [49] **Pakistani [65]**			Convenience of SB, e.g. TV	*n* = 1 **Pakistani [24]**				
				Ethnic minority concentration	*n* = 2 **General [37] Mixed [80]**	Concerns about safety	*n* = 1 **South Asian [54]**			Status of PE in some Muslim communities	*n* = 1 Muslim [20]				
				Habitus	*n* = 2 **Minorities [57] Mixed [68**]	Walking to school resisted	*n* = 1 **Mixed [29]**			Financial sanctions	*n* = 1 Multiethnic [47]				
				Social influence	*n* = 2 South Asian [52] **Turkish & Moroccan [53]**	Fear of falling	*n* = 1 South Asian [26]			Country	*n* = 1 Multiethnic [47]				
				Functional support	*n* = 2 South Asian [26, 52]	Lack of PA routine	*n* = 1 South Asian [26]			Structural barriers	*n* = 1 **South Asian [5]**				
				Facilitative relatives	*n* = 2 South Asian [26, 52]	Lack of intention	*n* = 1 Surinamese & Indian [23]			Crime	*n* = 1 **Turkish [59]**				
				Social network	*n* = 2 **Mixed [46, 72]**	Ability to use health care	*n* = 1 **Mixed [72]**								
				Preferred mode of transportation	*n* = 2 Immigrant [38] **Multiethnic [67]**	Body consciousness increased during adolescence	*n* = 1 Muslim [20]								
				Religious community	*n* = 2 **Diverse [45] Multiethnic [47]**	Religious consciousness increased during adolescence	*n* = 1 Muslim [20]								
				Stereotypes	*n* = 1 **Minority [42]**	Lack of PA skills	*n* = 1 **Pakistani [50]**								
				Parental marital status	*n* = 1 **General[37]**	Values associated with PA	*n* = 1 **Pakistani [50]**								
				Car use	*n* = 1 South Asian [34]	Behavioural control	*n* = 1 **Pakistani [65]**								
				Inactive parental lifestyle	*n* = 1 South Asian [34]	Identity	*n* = 1 **Pakistani [65]**								
				Increased sedentary activities	*n* = 1 South Asian [34]										
				Social resources	*n* = 1 **Minorities [60]**										
				Parents’ participation in organised sports	*n* = 1 **Minorities [60]**										
				Activities in own community	*n* = 1 South Asian [40]										
				Work ethics	*n* = 1 South Asian [40]										
				Historical experiences and adaptations	*n* = 1 **Minorities [57]**										
				Children as incentive to be active	*n* = 1 South Asian [27]										
				Partner’s or family disproval	*n* = 1 South Asian [27]										
				Behaviour of others	*n* = 1 **Turkish & Moroccan [53]**										
				Gym based exercise unfamiliar	*n* = 1 South Asian [52]										
				Collectivist norms	*n* = 1 South Asian [52]										
				Overprotective family	*n* = 1 South Asian [52]										
				Traditional authorities	*n* = 1 Surinamese & Indian [23]										
				Symbolic meaning of certain foods	*n* = 1 Surinamese & Indian [23]										
				Attitude of peers	*n* = 1 Musilim [18]										
				Traditional power relations	*n* = 1 Musilim [18]										

There were some similarities and differences in clusters of factors influencing PA and SB across different ethnic minority groups (Table 3). For example, “Social and cultural environment”, “Social and material resources” and “Migration context” were identified in many of the study populations and studies with mixed populations. Many of the factors in the clusters “Physical environment and accessibility”, “Psychosocial and institutional environment” and “Health and health communication” were common in studies conducted among South Asians, the largest group in the review, but also African, Caribbean, Turkish and mixed. Qualitative studies that focused on Muslim groups were represented in most clusters except “Migration context” and “Social and material resources”.

## Clusters

### “Social & cultural environment”

The “Social & cultural environment” cluster included most factors (55 factors). The most cited factor was gender [5, 18–39] (Table 2). Gender, as a factor that influences PA and SB in ethnic minority groups, span different notions, and included cultural and religious notions of gender, moralities related to gender, and as well as social expectations and gender roles.

Factors related to religion recurred often as well. These included: religious requirements [20, 26, 31, 34, 37, 40–43] and religion and culture [5, 18, 20, 26, 43–46]. Following the factors were, commitments within family [20, 24, 27, 31, 39, 40, 47, 48] and modesty [5, 23, 26, 27, 31, 38, 40, 49]. Other factors related to gender, family, religious and cultural issues were: women as caregiver/mother [5, 26, 27, 40, 42, 46, 48], ideals of behaviour [18, 24, 27–29, 34, 39], family [18, 23, 26, 28, 50–52] and shame [5, 23, 26, 27, 31, 38, 40]. The broader factors, religious requirements and religion and culture, encompassed religious requirements of dressing, as well as degree of religiosity or piety.

The cluster also included factors relating to cultural requirements [20, 24, 29, 34, 53, 54], social acceptance of PA [5, 22, 40, 54–56], social environment [27, 47, 49, 52, 57], social support [25, 26, 39, 53, 58], structural constraints in family [24, 31, 39, 59, 60] and concepts of age/aging [26, 27, 36, 52, 61]. Cultural requirements included ideals of behaviour and morality, as well as commitments of attending religious ceremonies and notions of the relationship between genders. The factor social acceptance of PA covered more specific factors. For instance, Babakus et al. [5] described that education about Muslim faith can be a motivating factor as PA is seen as central to the Muslim way of life.

Some studies included the factors: parental attitudes to PA [18, 20, 28, 60], lack of ‘exercise culture’ [29, 31, 52, 55], women not to be alone outside [28, 31, 54], purposeful PA selfish [5, 49, 54], age [20, 30, 58], religious festivals [19, 26, 27], religious prayer [19, 26, 27], peer group [26, 47, 52], few active friends or family [27, 42, 50], and PA role models [20, 23, 50]. The remaining factors were found in only one or two studies. Many studies were on South Asian and Muslim women, but also other groups.

### “Psychosocial”

The “Psychosocial” cluster included individually held factors. Knowledge of PA and health [4, 5, 19, 20, 23, 26, 40, 45, 50, 52, 54, 55, 61–64], the factor included in most studies, covers knowledge, notions and ideas about what constitutes PA and the relationship between health and PA. Self-image [20, 23, 26, 27, 47, 49, 50, 52, 53, 58, 65], which encompasses different notions of who one is and should be, was mainly reported in qualitative studies.

The cluster also included factors such as knowledge of PA [23, 26, 31, 40, 50, 55, 58, 62, 65], dangers of environment and strangers [5, 24, 26, 27, 31, 34, 45], ideas of ideal body [40, 41, 47, 54, 61, 62], confidence level [26, 49, 50, 53, 58, 65], prevent disease [5, 23, 25, 43, 58, 65], notions of karma/fatalism [5, 23, 31, 40, 52], experience of PA/PE [39, 47, 55, 58, 60], goal setting [23, 27, 40, 42, 50], motivation [26, 39, 40, 58, 65], notions of leisure-time PA [27, 31, 32, 66], lack of enjoyment of PA [23, 31, 45, 48], health beliefs about PA [23, 31, 54], attitudes [56, 61, 65], not the sporty type [27, 47, 65]. The remaining factors were found in only one or two articles. Study populations were mixed in relation to gender.

### “Physical environment & accessibility”

Sports facilities available [5, 18, 26, 27, 29, 31, 34, 45, 47, 50, 67], lack of appropriate activities [5, 18, 26, 27, 39, 40, 42, 61, 62, 65] and lack of culturally sensitive facilities [18, 19, 26, 27, 31, 39, 52] were the most widely cited PA and SB factors in this cluster. Lack of appropriate activities and lack of culturally sensitive facilities were often interwoven with gender issues, as for instance whether or not an activity was appropriate for women or whether women and men could exercise separately. Lack of women only facilities [18, 37, 39] was mentioned as a factor as well.

This cluster also included expenses [23, 27, 34, 41, 45, 61], religious dress requirements [18, 20, 22, 27, 38, 40], transportation and infrastructure [26, 27, 32, 47, 68], climate [23, 27, 31, 32, 69], unattractive neighbourhood [45, 47, 59, 68], lack of gym instructor support [23, 26, 52, 58], living in urban area [37, 40, 70], and home environment [32, 36, 47]. The remaining 20 factors were found in only one or two studies. Study populations were mixed in relation to gender.

### “Migration context”

The main factor in the “Migration context” cluster was language [25–27, 40, 49, 52, 54, 71, 72], meaning a lack or poor knowledge of the language of the host country. Language was seen as having an impact on PA and SB in several ways. As a barrier to get access of knowledge about PA, SB and health, as well as information on when and where to do PA. The second most cited factor in this cluster was acculturation [21, 30, 42, 53, 59, 72], meaning the ways minorities become part of the wider society. “Acculturation” was often related to other factors such as time since migration [21, 72–74], generation [57], and immigration history [37].

Other factors grouped under this cluster included country of birth [21, 44, 46, 71, 75] (implying cultural background, but also host country’s attitudes towards immigrants from a specific country), racial harassment [27, 40, 42, 54], and lack of knowledge of host culture [21, 54, 64]. The remaining seven factors were only found in only study each. Study populations were mixed in relation to gender.

### “Institutional environment”

The cluster “Institutional environment” included factors related to school and work environment. In schools, demands of curriculum [19, 29, 45] were seen as having an impact on PA both because schools prioritise other subjects than physical education (PE) [19], and because parents value homework over leisure time PA [45]. Head teacher’s attitude and resources [18–20] also had an impact on PA. Teachers could choose whether or not to let ethnic minority pupils participate on their own terms, i.e. separating boys and girls, or allowing ethnic dress requirements, and because limited resources can make it harder to prioritise PA or extra-curricular activities. Other factors included priorities in school [29, 41], exam pressure [20, 29], occupational PA [27, 59] and traditional livelihood [27, 66]. Many studies focused on Muslim women and girls.

### “Social and material resources”

Practicalities/responsibilities [24, 26–28, 31, 40, 42, 45, 52, 76] were the most cited factors assigned to the cluster “Social and material resources.” The cluster also consisted of time constraints [5, 27, 29, 31, 39, 40, 50], occupation [31, 39, 47, 67, 72], education [30, 39, 63, 67], financial limitations [5, 21, 39, 67], lack of parental time [24, 34, 41], taking time from family and gender role activities [5, 22, 31], and income [35, 47, 77]. The remaining four factors were only reported in one or two studies. Study populations were mixed in relation to gender.

### “Health and health communication”

This cluster included health conditions [23, 25–27, 31, 40, 47], physical health [26, 31, 49, 65], religious fasting [19, 26, 27], healthcare support [26, 49, 58], mental health [65, 70], pain [26, 49] and primary health care [47, 58]. The remaining five factors were found in one article. The majority of studies focused on women.

### “Political environment”

This is the cluster with the smallest number of factors: local political orientation [18–20], health care system adaptation [47, 58] and national political orientation [18]. Local and national political orientation were seen as having an impact on PA and SB in ethnic minority groups by the willingness to make adaptations to better suit minority populations. Health care system adaptation had similar impact on PA and SB, by conveying the message of PA and health in ways adapted to ethnic minority groups. Many studies focused on Muslim women and girls.

## Discussion

The main aims of this study were to identify factors influencing physical activity and sedentary behaviour among ethnic minority groups living in Europe, uncover gaps in the literature, and to suggest priorities for future research. The review extracted 183 factors that were grouped into eight clusters. The “Social & cultural environment” and “Psychosocial” were the clusters containing most factors.

An important finding of our study was that cultural and religious issues, in particular those related to gender issues, were recurring factors across the clusters. Among these were cultural ideas of the body. Ideas of healthy ways to move and appropriateness of exposing parts of the body to perform physical activity are culturally loaded and tend to vary according to age, gender and roles/responsibilities within the family. For instance, research among adolescents has shown that consciousness of one’s own body and religious consciousness evolve during these years, and affect individual’s involvement and meaning of PA [20]. Importantly, cultural and religious factors can both hinder and facilitate PA. For example, religious requirements on how to dress [18, 20, 22, 27, 38, 40] and limitations on spending time with the opposite gender might hinder PA, but staying fit and walking to religious sites for prayer were sometimes religious merits [27]. Other cultural issues involved ideas of femininity [43], religious or traditional ideas of ideal behaviour and attitudes related to leisure time. Moreover, there was the impact of lack of gender-segregated facilities [27, 39, 61]. Being female is not necessarily a barrier to PA in Muslim populations, but if there are no ways in which women can be active without encountering men, this might lead to women being less active. Not being able to separate between Islamic ideals (which is not against women being active) and particular cultural traditions of some Muslim populations (which might be counter to PA among women) was one factor of PA identified [20]. Therefore, understanding how religion and culture or tradition interact with other factors requires more in-depth study.

In stating that there are important factors to be found in the interchange between “gender,” “culture” and “religion,” it is vital to keep in mind that many of the studies have been undertaken among Muslim groups, thus the findings of this review reflect the populations studied.

The study showed that there were also many factors related to knowledge and information across the clusters, such as lack of knowledge and information about PA and the relationship between health and PA [4, 5, 20, 23, 26, 31, 40, 45, 50, 55, 58, 61–65]. Lack of knowledge and information about facilities in own community [31, 52, 77] were reported as well. Other emerging factors were lack of knowledge of new culture and familiarity with wider community [21, 54, 64].

Language capabilities [25–27, 40, 49, 52, 54, 71, 72] had impact on access to information and knowledge about opportunities for PA in local areas and recommendations for PA. Additionally, lack of fluency in language can make it harder to follow PA classes/courses.

The review indicated that ethnic minority groups are influenced by many of the same factors as majority populations, such as age, knowledge and physical environment [15, 78, 79], but the processes underlying these factors (culture, religion, lay models etc.) were distinctive for ethnic minority groups. Specific factors for ethnic minority groups, such as cultural, religious, and/or traditional values, perceptions and ideas (associated with PA, sedentary behaviour and body) emerged. The review showed that there are divergent notions of what constitutes physical activity and exercise [27, 31, 32, 66] and how different activities might relate to health [4, 5, 20, 26, 31, 40, 45, 50, 52, 54, 55, 61, 64]. Culturally dependent knowledge, notions and ideas of the physical activity and health and how they are related were among the most important reasons for not being active. For instance, some ethnic groups viewed physical activity as detrimental for health, especially for women and the elderly. Thus, it is important to gain knowledge about the cultural ideas of physical activity in different ethnic minority groups, but also to develop culturally sensitive information about the health benefits of physical activity, to create effective interventions and policies.

### Strengths and limitations of the review

This is the first systematic mapping review that has described factors influencing PA and SB among a diverse ethnic minority groups living in Europe. One difference between this review and earlier reviews is the method used in synthesizing the findings. In this review, we have used a novel categorization based on clustering factors. This approach transcends existing models by aiming at better capturing the complexity of the system of factors influencing behaviour [10, 13]. Another strength of the study is that the study design included both quantitative and qualitative published European literature. The review indicated that quantitative and qualitative studies contribute to our understanding of PA and SB among ethnic minority groups by providing somewhat different sets of factors. Qualitative studies used more explorative designs about PA and SB in ethnic groups, and yielded richer and more detailed information about the inter-relationship between factors and clusters. One challenge in this review is that the categorisation of ethnic groups varied widely between the different studies, making it difficult to compare the findings.

The review indicates that there is more evidence for the role of individual level factors like gender, knowledge of PA and health, and health conditions compared with environmental factors. This tendency might in part stem from the place of the individual in the “Western” society. However, it is also important to note that the number of studies citing each factor is not necessarily an indication of the importance of those factors. Rather, it tells us is how many studies have selected to focus on each factor. For instance, the fact that few factors related to political environment have been found does not mean that environmental factors are not important, but there has been less focus on them.

The importance of religion and gender issues may be due to the fact that many studies have been conducted on populations with Islamic faith. In order to further explore factors related to PA and SB among ethnic minorities, more studies on populations of different religious affiliation (for example, Hinduism, Catholicism) are needed.

### Implication of the findings

The literature review indicates that both PA and SB among ethnic minority groups in Europe are influenced by a wide variety of factors that are related and cut across different clusters of influence. Our findings support the need of adopting a systems-based framework [10] to capture the complexity of PA and SB among ethnic minority populations. Studies adopting a qualitative research design provided a richer understanding of underlying factors related to PA and SB.

The literature review indicates that comparative studies are limited. One recommendation from the review is that there is a need for systematic comparative research across Europe to shed light on the processes in which similar factors drive PA and SB behaviours on specific groups in different national and regional settings. Most studies included population from South Asia and Muslim populations, but as the European population is changing there is the need to further research on other ethnic groups, for example, asylum seeking groups from Somalia, Eritrea and Syria.

The majority of the studies were conducted in the Western part of Europe, mainly in the UK, and the Nordic countries. This was reflected in the groups included in the review, which do not represent the diversity of ethnic minority groups and religious populations living in Europe. This calls for a broadening of the research scope to include all parts of Europe.

Finally, the literature review indicates that there are very few studies on SB among ethnic minority groups in Europe. SB is a fairly new area of research and thus methods may not have not been adapted to ethnic minorities. As the relevance of SB for health outcomes is increasingly documented [15, 78], further research on this topic needs to specifically address ethnic minority groups.

## Conclusions

This systematic review identified 183 factors influencing PA and SB across some ethnic minority and religious groups (Muslim) living in Europe. Factors were grouped into eight clusters following a data driven approach. The most recurrent factors (gender, religion, cultural requirements and knowledge) were part of the clusters ‘Social & cultural environment’ and ‘Psychosocial’.

The review indicated that there are several gaps in the literature related to the ethnic minority populations studied, the countries where the studies have been conducted, paucity of comparative studies and lack of attention towards SB.

The review showed that there are some specific factors influencing PA and SB among ethnic minority groups. It is important to further address these factors in order to plan and implement effective interventions.

## Additional files

Additional file 1: Systematic search strategy. (DOCX 83 kb)

Additional file 2: Characteristics of quantitative and qualitative studies. (DOCX 17 kb)

## Abbreviations

DEDIPAC KH: Determinants of Diet and Physical Activity Knowledge Hub
NCDs: Non-communicable diseases
PA: Physical Activity
SB: Sedentary Behaviour
