# Supplementary material for: Systematic mapping review of the factors influencing physical activity and sedentary behaviour in ethnic minority groups in Europe: a DEDIPAC study
Source: Int J Behav Nutr Phys Act. 2017 Jul 24;14:99. doi: 10.1186/s12966-017-0554-3 (PMC5525226; doi:10.1186/s12966-017-0554-3)
Supplement: Supplementary file 1 — Systematic search strategy. (DOCX 83 kb) [file 12966_2017_554_MOESM1_ESM.docx]

## Additional file 1: Systematic search strategy

Searches for review 2.4 September 3^rd^ 2014

Web of science: 5084

| # 10 | [5,084](http://apps.webofknowledge.com/summary.do?product=WOS&doc=1&qid=28&SID=Q1LtjuPjGOiuJl1cGCC&search_mode=CombineSearches) | #9 AND #8 AND #3  Indexes=SCI-EXPANDED, SSCI, A&HCI Timespan=1999-2014 | [Edit](http://apps.webofknowledge.com/WOS_AdvancedSearch_input.do?product=WOS&SID=Q1LtjuPjGOiuJl1cGCC&search_mode=AdvancedSearch&replaceSetId=10&editState=init) | Select to combine sets.  | Select to delete this set.  |
| --- | --- | --- | --- | --- | --- |
|  | | | | | |
| # 9 | [85,607](http://apps.webofknowledge.com/summary.do?product=WOS&doc=1&qid=27&SID=Q1LtjuPjGOiuJl1cGCC&search_mode=CombineSearches) | #6 OR #5  Indexes=SCI-EXPANDED, SSCI, A&HCI Timespan=1999-2014 | [Edit](http://apps.webofknowledge.com/WOS_AdvancedSearch_input.do?product=WOS&SID=Q1LtjuPjGOiuJl1cGCC&search_mode=AdvancedSearch&replaceSetId=9&editState=init) | Select to combine sets.  | Select to delete this set.  |
|  | | | | | |
| # 8 | [9,358,339](http://apps.webofknowledge.com/summary.do?product=WOS&doc=1&qid=26&SID=Q1LtjuPjGOiuJl1cGCC&search_mode=CombineSearches) | #7 OR #4 OR #2  Indexes=SCI-EXPANDED, SSCI, A&HCI Timespan=1999-2014 | [Edit](http://apps.webofknowledge.com/WOS_AdvancedSearch_input.do?product=WOS&SID=Q1LtjuPjGOiuJl1cGCC&search_mode=AdvancedSearch&replaceSetId=8&editState=init) | Select to combine sets.  | Select to delete this set.  |
|  | | | | | |
| # 7 | [7,222,035](http://apps.webofknowledge.com/summary.do?product=WOS&doc=1&qid=25&SID=Q1LtjuPjGOiuJl1cGCC&search_mode=GeneralSearch) | **TOPIC:** (pala* or panam*or papua new guinea or paragu* or per* or phillipen* or rwand* or samo* or sao tome* and principe* or Senegal* or Seychelles or sierra leon* or solomon islands or Somali* or south afric* or south suda* or sri lank* or st luc* or st vincen* or suda* or surina* or Swaziland or Syrian arab republic or tajikista* or tanzan* or thail* or "timoe leste*" or tog* or tong* or turkmensista* or tuval* or ugand* or uzbekist* or vanuat* or venezuel* or vietna* or west bank and gaza or yemen* or zambi* or zimbabw*.ti,ab.)  Indexes=SCI-EXPANDED, SSCI, A&HCI Timespan=1999-2014 | [Edit](http://apps.webofknowledge.com/WOS_AdvancedSearch_input.do?product=WOS&SID=Q1LtjuPjGOiuJl1cGCC&search_mode=AdvancedSearch&replaceSetId=7&editState=init) | Select to combine sets.  | Select to delete this set.  |
|  | | | | | |
| # 6 | [83,918](http://apps.webofknowledge.com/summary.do?product=WOS&doc=1&qid=23&SID=Q1LtjuPjGOiuJl1cGCC&search_mode=GeneralSearch) | **TOPIC:** (*"physical activit*"/ or *"motor activit*"/ or excerci*.mp. or movement.mp. or sedentary.mp. or lifestyle*.mp. or behavio*.mp. or inactivt*.mp. or walk*.mp. or sitt*.ti,ab.)  Indexes=SCI-EXPANDED, SSCI, A&HCI Timespan=1999-2014 | [Edit](http://apps.webofknowledge.com/WOS_AdvancedSearch_input.do?product=WOS&SID=Q1LtjuPjGOiuJl1cGCC&search_mode=AdvancedSearch&replaceSetId=6&editState=init) | Select to combine sets.  | Select to delete this set.  |
|  | | | | | |
| # 5 | [85,208](http://apps.webofknowledge.com/summary.do?product=WOS&doc=1&qid=22&SID=Q1LtjuPjGOiuJl1cGCC&search_mode=GeneralSearch) | **TOPIC:** (*"Motor Activity"/ or "Walking".mp. or "Movement".mp. or *"Sedentary Lifestyle"/ or *"physical activit*"/ or *"sedentary behavio*"/)  Indexes=SCI-EXPANDED, SSCI, A&HCI Timespan=1999-2014 | [Edit](http://apps.webofknowledge.com/WOS_AdvancedSearch_input.do?product=WOS&SID=Q1LtjuPjGOiuJl1cGCC&search_mode=AdvancedSearch&replaceSetId=5&editState=init) | Select to combine sets.  | Select to delete this set.  |
|  | | | | | |
| # 4 | [2,295](http://apps.webofknowledge.com/summary.do?product=WOS&doc=1&qid=19&SID=Q1LtjuPjGOiuJl1cGCC&search_mode=GeneralSearch) | **TOPIC:** (((((((*chinese*/ or *china/ or *irish traveller*/ or gyps*.mp. or *roma/ or *Asian/ or *Pakistan*/ or *banglades*/ or *turk*/ or *iran/ or *Iranian*/ or *irak*/ or *Maghreb*/ or *tunisi*/ or *morocco*/ or *liby*/ or *algeri*/ or *Surinam*/ or *latin American*/ or *cameroo*/ or *equado*/ or *eritre*/ or *chil*/ or *estoni*/ or *latvi*/ or *lithuani*/ or *goergi*/ or *armeni*/ or *azerbaija*/ or *kazakhsta*/ or *ukrain*/ or *belaru*/ or *moldov*/ or *croati*/ or *Czech republic*/ or *pol*/ or *romani*/ or *serbi*/ or *slovaki*/ or *sloveni*/ or *albani*/ or *bulgari*/ or *macedoni/ or *montenegr*/ or *angol*/ or *beliz*/ or *beni*/ or *bhuta*/ or *boliv*/ or *botswan*/ or *brazil*/ or *Burkina faso/ or *burund*/ or *chad/ or *colombi*/ or *cong*/ or *costa ric*/ or *ivoria*/ or *comor*/ or *cub*/ or *White/) and Black Caribbean.mp.) or *white/) and Black African.mp.) or *White/) and Asian.mp.) or *Mixed ethnic background/ or *multiple ethnic background*/ or *Central African Republic/ or djibout*.mp. or domini*.mp. or equado*.mp. or egyp*.mp. or ethiop*.mp. or fij*.mp. or gabo*.mp. or gambit*.mp. or ghan*.mp. or grenad*.mp. or guatemal*.mp. or guine*.mp. or guinea Bissau.mp. or guyan*.mp. or hait*.mp. or hondura*.mp. or hungar*.mp. or indones*.mp. or ira*.mp. or jamaic*.mp. or jord*.mp. or keny*.mp. or kiribat*.mp. or kore*.mp. or koso*.mp. or leban*.mp. or lesot*.mp. or liber*.mp. or macedon*.mp. or madagassc*.mp. or malaw*.mp. or lao pdr.mp. or malays*.mp. or mald*.mp. or marshall island*.mp. or maurit*.mp. or mexic*.mp. or micronesi*.mp. or moldov*.mp. or mongol*.mp. or mozambi*.mp. or myanma*.mp. or namib*.mp. or nep*.mp. or nicaragu*.mp. or nig*.mp. or nigeri*.mp. or Dominican republic.mp. or el-salvado*.mp. or kyrgyz republic.ti,ab.)  Indexes=SCI-EXPANDED, SSCI, A&HCI Timespan=1999-2014 | [Edit](http://apps.webofknowledge.com/WOS_AdvancedSearch_input.do?product=WOS&SID=Q1LtjuPjGOiuJl1cGCC&search_mode=AdvancedSearch&replaceSetId=4&editState=init) | Select to combine sets.  | Select to delete this set.  |
|  | | | | | |
| # 3 | [960,063](http://apps.webofknowledge.com/summary.do?product=WOS&doc=1&qid=17&SID=Q1LtjuPjGOiuJl1cGCC&search_mode=GeneralSearch) | **TOPIC:** (Europe/ or *Russia/ or *Ukraine/ or *France/ or *Spain/ or *Sweden/ or *Norway/ or *Germany/ or *Finland/ or *Poland/ or *Italy/ or *Great Britain/ or *Romania/ or *"Republic of Belarus"/ or *Kazakhstan/ or *Greece/ or *Bulgaria/ or *Iceland/ or *Hungary/ or *Portugal/ or *Austria/ or *Czech Republic/ or *Serbia/ or *Ireland/ or *Latvia/ or *Bosnia-Herzegovina/ or *Croatia/ or *Lithuania/ or *Slovakia/ or *Estonia/ or *Denmark/ or *Netherlands/ or *Switzerland/ or *Moldova/ or *Belgium/ or *Albania/ or *"Macedonia (Republic)"/ or *Turkey/ or *Slovenia/ or *Montenegro/ or *cyprus/ or *malta/ or *Azerbaijan/ or *Luxembourg/ or *Georgia/ or *Andorra/ or *Liechtenstein/ or *Monaco/ or *Vatican City/ or *San Marino/ or (Europ or Russia or Ukraine or France or Spain or Sweden or Norway or Germany or (Finland or Poland or Italy or United Kingdom or Great Britain or Romania or Belarus) or (Kazakhstan or Greece or Bulgaria or Iceland or Hungary or Portugal or Austria or Czech Republic or Serbia or Republic of Ireland or Latvia) or (Lithuania or Croatia or Slovakia or Estonia or Denmark or Netherlands or Switzerland or Moldova or Belgium or Albania or Macedonia or Turkey or Slovenia or Montenegro or Cyprus or Azerbaijan or Luxembourg or Georgia or Andorra or Malta or Liechtenstein or San Marino or Monaco or Vatican city) or (Bosnia adj1 Herzegovina)).ti,ab.)  Indexes=SCI-EXPANDED, SSCI, A&HCI Timespan=1999-2014 | [Edit](http://apps.webofknowledge.com/WOS_AdvancedSearch_input.do?product=WOS&SID=Q1LtjuPjGOiuJl1cGCC&search_mode=AdvancedSearch&replaceSetId=3&editState=init) | Select to combine sets.  | Select to delete this set.  |
|  | | | | | |
| # 2 | [4,091,470](http://apps.webofknowledge.com/summary.do?product=WOS&doc=1&qid=16&SID=Q1LtjuPjGOiuJl1cGCC&search_mode=GeneralSearch) | **TOPIC:** (*"Emigrants and Immigrants" or *"cultural diversity" or *"Minority Groups" or *"Transients and Migrants" or *"Ethnic Groups" or multiculturalism or "ethnic minorit*" or BME or "black minorit*" or ethnic* or asylum seeker* or refugee* or "african caribbean*" or *West Indies or *Afro-caribbean* or *Non-white or Coloured population or Black* or Afric* or Indi* or Caucasian* or Caribbean* or Arab* or "Black Afric*" or "South Asia*" or "Trinidad and Tobago" or emigrant* or immigrant* or minorit* or migrant* or minorit*)  Indexes=SCI-EXPANDED, SSCI, A&HCI Timespan=1999-2014 | [Edit](http://apps.webofknowledge.com/WOS_AdvancedSearch_input.do?product=WOS&SID=Q1LtjuPjGOiuJl1cGCC&search_mode=AdvancedSearch&replaceSetId=2&editState=init) | Select to combine sets.  | Select to delete this set.  |
|  | | | | | |
| # 1 | [4,091,470](http://apps.webofknowledge.com/summary.do?product=WOS&doc=1&qid=15&SID=Q1LtjuPjGOiuJl1cGCC&search_mode=GeneralSearch) | **TOPIC:** (*"Emigrants and Immigrants" or *"cultural diversity" or *"Minority Groups" or *"Transients and Migrants" or *"Ethnic Groups" or multiculturalism or "ethnic minorit*" or BME or "black minorit*" or ethnic* or asylum seeker* or refugee* or "african caribbean*" or *West Indies or *Afro-caribbean* or *Non-white or Coloured population or Black* or Afric* or Indi* or Caucasian* or Caribbean* or Arab* or "Black Afric*" or "South Asia*" or "Trinidad and Tobago" or emigrant* or immigrant* or minorit* or migrant* or minorit*)  Indexes=SCI-EXPANDED, SSCI, A&HCI Timespan=1999-2014 |  |  |  |

Searcing articles and titles/abstracts only: 3786

| # 9 | [3,789](http://apps.webofknowledge.com/summary.do?product=WOS&doc=1&qid=53&SID=Q1LtjuPjGOiuJl1cGCC&search_mode=CombineSearches) | #8 AND #6 AND #2  Indexes=SCI-EXPANDED, SSCI, A&HCI Timespan=1999-2014 | [Edit](http://apps.webofknowledge.com/WOS_AdvancedSearch_input.do?product=WOS&SID=Q1LtjuPjGOiuJl1cGCC&search_mode=AdvancedSearch&replaceSetId=9&editState=init) | Select to combine sets.  | Select to delete this set.  |
| --- | --- | --- | --- | --- | --- |
|  | | | | | |
| # 8 | [66,915](http://apps.webofknowledge.com/summary.do?product=WOS&doc=1&qid=52&SID=Q1LtjuPjGOiuJl1cGCC&search_mode=CombineSearches) | #7 OR #1  Indexes=SCI-EXPANDED, SSCI, A&HCI Timespan=1999-2014 | [Edit](http://apps.webofknowledge.com/WOS_AdvancedSearch_input.do?product=WOS&SID=Q1LtjuPjGOiuJl1cGCC&search_mode=AdvancedSearch&replaceSetId=8&editState=init) | Select to combine sets.  | Select to delete this set.  |
|  | | | | | |
| # 7 | [6](http://apps.webofknowledge.com/summary.do?product=WOS&doc=1&qid=44&SID=Q1LtjuPjGOiuJl1cGCC&search_mode=AdvancedSearch) | (TS=((*"Motor Activity"/ or "Walking" or "Movement" or *"Sedentary Lifestyle"/ or *"physical activit*"/ or *"sedentary behavio*"/).ti,ab.)) *AND* **DOCUMENT TYPES:** (Article)  Indexes=SCI-EXPANDED, SSCI, A&HCI Timespan=1999-2014 | [Edit](http://apps.webofknowledge.com/WOS_AdvancedSearch_input.do?product=WOS&SID=Q1LtjuPjGOiuJl1cGCC&search_mode=AdvancedSearch&replaceSetId=7&editState=init) | Select to combine sets.  | Select to delete this set.  |
|  | | | | | |
| # 6 | [6,527,131](http://apps.webofknowledge.com/summary.do?product=WOS&doc=1&qid=41&SID=Q1LtjuPjGOiuJl1cGCC&search_mode=CombineSearches) | #5 OR #4 OR #3  Indexes=SCI-EXPANDED, SSCI, A&HCI Timespan=1999-2014 | [Edit](http://apps.webofknowledge.com/WOS_AdvancedSearch_input.do?product=WOS&SID=Q1LtjuPjGOiuJl1cGCC&search_mode=AdvancedSearch&replaceSetId=6&editState=init) | Select to combine sets.  | Select to delete this set.  |
|  | | | | | |
| # 5 | [6,526,025](http://apps.webofknowledge.com/summary.do?product=WOS&doc=1&qid=40&SID=Q1LtjuPjGOiuJl1cGCC&search_mode=AdvancedSearch) | (TS=(pala* or panam*or papua new guinea or paragu* or per* or phillipen* or rwand* or samo* or sao tome* and principe* or Senegal* or Seychelles or sierra leon* or solomon islands or Somali* or south afric* or south suda* or sri lank* or st luc* or st vincen* or suda* or surina* or Swaziland or Syrian arab republic or tajikista* or tanzan* or thail* or "timoe leste*" or tog* or tong* or turkmensista* or tuval* or ugand* or uzbekist* or vanuat* or venezuel* or vietna* or west bank and gaza or yemen* or zambi* or zimbabw*.ti,ab.)) *AND* **DOCUMENT TYPES:** (Article)  Indexes=SCI-EXPANDED, SSCI, A&HCI Timespan=1999-2014 | [Edit](http://apps.webofknowledge.com/WOS_AdvancedSearch_input.do?product=WOS&SID=Q1LtjuPjGOiuJl1cGCC&search_mode=AdvancedSearch&replaceSetId=5&editState=init) | Select to combine sets.  | Select to delete this set.  |
|  | | | | | |
| # 4 | [437](http://apps.webofknowledge.com/summary.do?product=WOS&doc=1&qid=39&SID=Q1LtjuPjGOiuJl1cGCC&search_mode=AdvancedSearch) | (TS=((*"Emigrants and Immigrants" or *"cultural diversity" or *"Minority Groups" or *"Transients and Migrants" or *"Ethnic Groups" or multiculturalism or "ethnic minorit*" or BME or "black minorit*" or ethnic* or asylum seeker* or refugee* or "african caribbean*" or *West Indies or *Afro-caribbean* or *Non-white or Coloured population or Black* or Afric* or Indi* or Caucasian* or Caribbean* or Arab* or "Black Afric*" or "South Asia*" or "Trinidad and Tobago" or emigrant* or immigrant* or minorit* or migrant* or minorit*).ti,ab.)) *AND* **DOCUMENT TYPES:** (Article)  Indexes=SCI-EXPANDED, SSCI, A&HCI Timespan=1999-2014 | [Edit](http://apps.webofknowledge.com/WOS_AdvancedSearch_input.do?product=WOS&SID=Q1LtjuPjGOiuJl1cGCC&search_mode=AdvancedSearch&replaceSetId=4&editState=init) | Select to combine sets.  | Select to delete this set.  |
|  | | | | | |
| # 3 | [2,115](http://apps.webofknowledge.com/summary.do?product=WOS&doc=1&qid=37&SID=Q1LtjuPjGOiuJl1cGCC&search_mode=AdvancedSearch) | (TS=(((((((*chinese*/ or *china/ or *irish traveller*/ or gyps*.mp. or *roma/ or *Asian/ or *Pakistan*/ or *banglades*/ or *turk*/ or *iran/ or *Iranian*/ or *irak*/ or *Maghreb*/ or *tunisi*/ or *morocco*/ or *liby*/ or *algeri*/ or *Surinam*/ or *latin American*/ or *cameroo*/ or *equado*/ or *eritre*/ or *chil*/ or *estoni*/ or *latvi*/ or *lithuani*/ or *goergi*/ or *armeni*/ or *azerbaija*/ or *kazakhsta*/ or *ukrain*/ or *belaru*/ or *moldov*/ or *croati*/ or *Czech republic*/ or *pol*/ or *romani*/ or *serbi*/ or *slovaki*/ or *sloveni*/ or *albani*/ or *bulgari*/ or *macedoni/ or *montenegr*/ or *angol*/ or *beliz*/ or *beni*/ or *bhuta*/ or *boliv*/ or *botswan*/ or *brazil*/ or *Burkina faso/ or *burund*/ or *chad/ or *colombi*/ or *cong*/ or *costa ric*/ or *ivoria*/ or *comor*/ or *cub*/ or *White/) and Black Caribbean.mp.) or *white/) and Black African.mp.) or *White/) and Asian.mp.) or *Mixed ethnic background/ or *multiple ethnic background*/ or *Central African Republic/ or djibout*.mp. or domini*.mp. or equado*.mp. or egyp*.mp. or ethiop*.mp. or fij*.mp. or gabo*.mp. or gambit*.mp. or ghan*.mp. or grenad*.mp. or guatemal*.mp. or guine*.mp. or guinea Bissau.mp. or guyan*.mp. or hait*.mp. or hondura*.mp. or hungar*.mp. or indones*.mp. or ira*.mp. or jamaic*.mp. or jord*.mp. or keny*.mp. or kiribat*.mp. or kore*.mp. or koso*.mp. or leban*.mp. or lesot*.mp. or liber*.mp. or macedon*.mp. or madagassc*.mp. or malaw*.mp. or lao pdr.mp. or malays*.mp. or mald*.mp. or marshall island*.mp. or maurit*.mp. or mexic*.mp. or micronesi*.mp. or moldov*.mp. or mongol*.mp. or mozambi*.mp. or myanma*.mp. or namib*.mp. or nep*.mp. or nicaragu*.mp. or nig*.mp. or nigeri*.mp. or Dominican republic.mp. or el-salvado*.mp. or kyrgyz republic.ti,ab.)) *AND* **DOCUMENT TYPES:** (Article)  Indexes=SCI-EXPANDED, SSCI, A&HCI Timespan=1999-2014 | [Edit](http://apps.webofknowledge.com/WOS_AdvancedSearch_input.do?product=WOS&SID=Q1LtjuPjGOiuJl1cGCC&search_mode=AdvancedSearch&replaceSetId=3&editState=init) | Select to combine sets.  | Select to delete this set.  |
|  | | | | | |
| # 2 | [788,371](http://apps.webofknowledge.com/summary.do?product=WOS&doc=1&qid=36&SID=Q1LtjuPjGOiuJl1cGCC&search_mode=AdvancedSearch) | (TS=(Europe/ or *Russia/ or *Ukraine/ or *France/ or *Spain/ or *Sweden/ or *Norway/ or *Germany/ or *Finland/ or *Poland/ or *Italy/ or *Great Britain/ or *Romania/ or *"Republic of Belarus"/ or *Kazakhstan/ or *Greece/ or *Bulgaria/ or *Iceland/ or *Hungary/ or *Portugal/ or *Austria/ or *Czech Republic/ or *Serbia/ or *Ireland/ or *Latvia/ or *Bosnia-Herzegovina/ or *Croatia/ or *Lithuania/ or *Slovakia/ or *Estonia/ or *Denmark/ or *Netherlands/ or *Switzerland/ or *Moldova/ or *Belgium/ or *Albania/ or *"Macedonia (Republic)"/ or *Turkey/ or *Slovenia/ or *Montenegro/ or *cyprus/ or *malta/ or *Azerbaijan/ or *Luxembourg/ or *Georgia/ or *Andorra/ or *Liechtenstein/ or *Monaco/ or *Vatican City/ or *San Marino/ or (Europ or Russia or Ukraine or France or Spain or Sweden or Norway or Germany or (Finland or Poland or Italy or United Kingdom or Great Britain or Romania or Belarus) or (Kazakhstan or Greece or Bulgaria or Iceland or Hungary or Portugal or Austria or Czech Republic or Serbia or Republic of Ireland or Latvia) or (Lithuania or Croatia or Slovakia or Estonia or Denmark or Netherlands or Switzerland or Moldova or Belgium or Albania or Macedonia or Turkey or Slovenia or Montenegro or Cyprus or Azerbaijan or Luxembourg or Georgia or Andorra or Malta or Liechtenstein or San Marino or Monaco or Vatican city) or (Bosnia adj1 Herzegovina)).ti,ab.)) *AND* **DOCUMENT TYPES:** (Article)  Indexes=SCI-EXPANDED, SSCI, A&HCI Timespan=1999-2014 | [Edit](http://apps.webofknowledge.com/WOS_AdvancedSearch_input.do?product=WOS&SID=Q1LtjuPjGOiuJl1cGCC&search_mode=AdvancedSearch&replaceSetId=2&editState=init) | Select to combine sets.  | Select to delete this set.  |
|  | | | | | |
| # 1 | [66,909](http://apps.webofknowledge.com/summary.do?product=WOS&doc=1&qid=45&SID=Q1LtjuPjGOiuJl1cGCC&search_mode=AdvancedSearch) | (TS=(*"physical activit*"/ or *"motor activit*"/ or excerci*.mp. or movement.mp. or sedentary.mp. or lifestyle*.mp. or behavio*.mp. or inactivt*.mp. or walk*.mp. or sitt*.ti,ab.)) *AND* **DOCUMENT TYPES:** (Article)  Indexes=SCI-EXPANDED, SSCI, A&HCI Timespan=1999-2014 |  |  |  |

OVID : 159

| 16 | ("Emigrants and Immigrants" or "cultural diversity" or "Minority Groups" or "Transients and Migrants" or "Ethnic Groups" or multiculturalism or "ethnic minorit*" or BME or "black minorit*" or ethnic* or asylum seeker* or refugee* or "african caribbean*" or West Indies or Afro-caribbean* or Non-white or Coloured population or Black* or Afric* or Indi* or Caucasian* or Caribbean* or Arab* or "Black Afric*" or "South Asia*" or "Trinidad and Tobago" or emigrant* or immigrant* or minorit* or migrant* or minorit*).kw,ti. | 539419 | Advanced | [[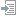](http://ovidsp.uk.ovid.com/sp-3.13.0b/ovidweb.cgi?&S=BMAGPDADOMHFJPCEFNLKGHBGPBPKAA00&SELECT=S.sh\|&R=16&Process+Action=display)Display](http://ovidsp.uk.ovid.com/sp-3.13.0b/ovidweb.cgi?&S=BMAGPDADOMHFJPCEFNLKGHBGPBPKAA00&SELECT=S.sh%7c&R=16&Process+Action=display)  [[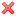](http://ovidsp.uk.ovid.com/sp-3.13.0b/ovidweb.cgi?&S=BMAGPDADOMHFJPCEFNLKGHBGPBPKAA00&SELECT=S.sh\|&R=16&Process+Action=delete)Delete](http://ovidsp.uk.ovid.com/sp-3.13.0b/ovidweb.cgi?&S=BMAGPDADOMHFJPCEFNLKGHBGPBPKAA00&SELECT=S.sh%7c&R=16&Process+Action=delete)  [[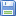](http://ovidsp.uk.ovid.com/sp-3.13.0b/ovidweb.cgi?&S=BMAGPDADOMHFJPCEFNLKGHBGPBPKAA00&SELECT=S.sh\|&R=16&Process+Action=save)Save](http://ovidsp.uk.ovid.com/sp-3.13.0b/ovidweb.cgi?&S=BMAGPDADOMHFJPCEFNLKGHBGPBPKAA00&SELECT=S.sh%7c&R=16&Process+Action=save)  [[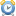](http://ovidsp.uk.ovid.com/sp-3.13.0b/ovidweb.cgi?&S=BMAGPDADOMHFJPCEFNLKGHBGPBPKAA00&SELECT=S.sh\|&R=16&Process+Action=autoalert)Auto-Alert](http://ovidsp.uk.ovid.com/sp-3.13.0b/ovidweb.cgi?&S=BMAGPDADOMHFJPCEFNLKGHBGPBPKAA00&SELECT=S.sh%7c&R=16&Process+Action=autoalert)  [[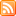](http://ovidsp.uk.ovid.com/sp-3.13.0b/ovidweb.cgi?&S=BMAGPDADOMHFJPCEFNLKGHBGPBPKAA00&SELECT=S.sh\|&R=16&Process+Action=rss_feed)RSS Feed](http://ovidsp.uk.ovid.com/sp-3.13.0b/ovidweb.cgi?&S=BMAGPDADOMHFJPCEFNLKGHBGPBPKAA00&SELECT=S.sh%7c&R=16&Process+Action=rss_feed) |  |
| --- | --- | --- | --- | --- | --- |
|  | 17 | ("Motor Activity" or "Walking" or "Movement" or "Sedentary Lifestyle" or "physical activity" or "sedentary behavio*").kw,ti. | 91028 | Advanced | [[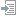](http://ovidsp.uk.ovid.com/sp-3.13.0b/ovidweb.cgi?&S=BMAGPDADOMHFJPCEFNLKGHBGPBPKAA00&SELECT=S.sh\|&R=17&Process+Action=display)Display](http://ovidsp.uk.ovid.com/sp-3.13.0b/ovidweb.cgi?&S=BMAGPDADOMHFJPCEFNLKGHBGPBPKAA00&SELECT=S.sh%7c&R=17&Process+Action=display)  [More ≫](http://ovidsp.uk.ovid.com/sp-3.13.0b/ovidweb.cgi) |
|  | 18 | (((((((chinese* or china or irish traveller* or gyps* or roma or Asian or Pakistan* or banglades* or turk* or iran or Iranian* or irak* or Maghreb* or tunisi* or morocco* or liby* or algeri* or Surinam* or latin American* or cameroo* or equado* or eritre* or chil* or estoni* or latvi* or lithuani* or goergi* or armeni* or azerbaija* or kazakhsta* or ukrain* or belaru* or moldov* or croati* or Czech republic* or pol* or romani* or serbi* or slovaki* or sloveni* or albani* or bulgari* or macedoni or montenegr* or angol* or beliz* or beni* or bhuta* or boliv* or botswan* or brazil* or Burkina faso or burund* or chad or colombi* or cong* or costa ric* or ivoria* or comor* or cub* or White) and Black Caribbean) or white) and Black African) or White) and Asian) or "Mixed ethnic background" or "multiple ethnic background" or Central African Republic or djibout* or domini* or equado* or egyp* or ethiop* or fij* or gabo* or gambit* or ghan* or grenad* or guatemal* or guine* or guinea Bissau or guyan* or hait* or hondura* or hungar* or indones* or ira* or jamaic* or jord* or keny* or kiribat* or kore* or koso* or leban* or lesot* or liber* or macedon* or madagassc* or malaw* or lao or malays* or mald* or marshall island* or maurit* or mexic* or micronesi* or moldov* or mongol* or mozambi* or myanma* or namib* or nep* or nicaragu* or nig* or nigeri* or Dominican republic or el-salvado* or kyrgyz republic).kw,ti. | 412352 | Advanced | [[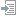](http://ovidsp.uk.ovid.com/sp-3.13.0b/ovidweb.cgi?&S=BMAGPDADOMHFJPCEFNLKGHBGPBPKAA00&SELECT=S.sh\|&R=18&Process+Action=display)Display](http://ovidsp.uk.ovid.com/sp-3.13.0b/ovidweb.cgi?&S=BMAGPDADOMHFJPCEFNLKGHBGPBPKAA00&SELECT=S.sh%7c&R=18&Process+Action=display)  [[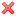](http://ovidsp.uk.ovid.com/sp-3.13.0b/ovidweb.cgi?&S=BMAGPDADOMHFJPCEFNLKGHBGPBPKAA00&SELECT=S.sh\|&R=18&Process+Action=delete)Delete](http://ovidsp.uk.ovid.com/sp-3.13.0b/ovidweb.cgi?&S=BMAGPDADOMHFJPCEFNLKGHBGPBPKAA00&SELECT=S.sh%7c&R=18&Process+Action=delete)  [[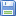](http://ovidsp.uk.ovid.com/sp-3.13.0b/ovidweb.cgi?&S=BMAGPDADOMHFJPCEFNLKGHBGPBPKAA00&SELECT=S.sh\|&R=18&Process+Action=save)Save](http://ovidsp.uk.ovid.com/sp-3.13.0b/ovidweb.cgi?&S=BMAGPDADOMHFJPCEFNLKGHBGPBPKAA00&SELECT=S.sh%7c&R=18&Process+Action=save)  [[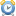](http://ovidsp.uk.ovid.com/sp-3.13.0b/ovidweb.cgi?&S=BMAGPDADOMHFJPCEFNLKGHBGPBPKAA00&SELECT=S.sh\|&R=18&Process+Action=autoalert)Auto-Alert](http://ovidsp.uk.ovid.com/sp-3.13.0b/ovidweb.cgi?&S=BMAGPDADOMHFJPCEFNLKGHBGPBPKAA00&SELECT=S.sh%7c&R=18&Process+Action=autoalert)  [[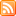](http://ovidsp.uk.ovid.com/sp-3.13.0b/ovidweb.cgi?&S=BMAGPDADOMHFJPCEFNLKGHBGPBPKAA00&SELECT=S.sh\|&R=18&Process+Action=rss_feed)RSS Feed](http://ovidsp.uk.ovid.com/sp-3.13.0b/ovidweb.cgi?&S=BMAGPDADOMHFJPCEFNLKGHBGPBPKAA00&SELECT=S.sh%7c&R=18&Process+Action=rss_feed) |
|  | 19 | (Europe or Russia or Finland or Poland or Italy or Great Britain or Romania or "Republic of Belarus" or Kazakhstan or Greece or Bulgaria or Iceland or Hungary or Portugal or Austria or Czech Republic or Serbia or Ireland or Latvia or Bosnia-Herzegovina or Croatia or Lithuania or Slovakia or Estonia or Denmark or Netherlands or Switzerland or Moldova or Belgium or Albania or "Macedonia (Republic)" or Malta or Ukraine or France or Spain or Sweden or Norway or Germany or United Kingdom or Belarus or Republic of Ireland or Latvia or Lithuania or Croatia or Slovakia or Estonia or Denmark or Netherlands or Switzerland or Moldova or Belgium or Albania or Macedonia or Turkey or Slovenia or Montenegro or Cyprus or Azerbaijan or Luxembourg or Georgia or Andorra or Malta or Liechtenstein or San Marino or Monaco or Vatican city or Bosnia Herzegovina).kw,ti. | 254974 | Advanced | [[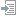](http://ovidsp.uk.ovid.com/sp-3.13.0b/ovidweb.cgi?&S=BMAGPDADOMHFJPCEFNLKGHBGPBPKAA00&SELECT=S.sh\|&R=19&Process+Action=display)Display](http://ovidsp.uk.ovid.com/sp-3.13.0b/ovidweb.cgi?&S=BMAGPDADOMHFJPCEFNLKGHBGPBPKAA00&SELECT=S.sh%7c&R=19&Process+Action=display)  [[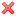](http://ovidsp.uk.ovid.com/sp-3.13.0b/ovidweb.cgi?&S=BMAGPDADOMHFJPCEFNLKGHBGPBPKAA00&SELECT=S.sh\|&R=19&Process+Action=delete)Delete](http://ovidsp.uk.ovid.com/sp-3.13.0b/ovidweb.cgi?&S=BMAGPDADOMHFJPCEFNLKGHBGPBPKAA00&SELECT=S.sh%7c&R=19&Process+Action=delete)  [[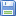](http://ovidsp.uk.ovid.com/sp-3.13.0b/ovidweb.cgi?&S=BMAGPDADOMHFJPCEFNLKGHBGPBPKAA00&SELECT=S.sh\|&R=19&Process+Action=save)Save](http://ovidsp.uk.ovid.com/sp-3.13.0b/ovidweb.cgi?&S=BMAGPDADOMHFJPCEFNLKGHBGPBPKAA00&SELECT=S.sh%7c&R=19&Process+Action=save)  [[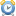](http://ovidsp.uk.ovid.com/sp-3.13.0b/ovidweb.cgi?&S=BMAGPDADOMHFJPCEFNLKGHBGPBPKAA00&SELECT=S.sh\|&R=19&Process+Action=autoalert)Auto-Alert](http://ovidsp.uk.ovid.com/sp-3.13.0b/ovidweb.cgi?&S=BMAGPDADOMHFJPCEFNLKGHBGPBPKAA00&SELECT=S.sh%7c&R=19&Process+Action=autoalert)  [[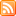](http://ovidsp.uk.ovid.com/sp-3.13.0b/ovidweb.cgi?&S=BMAGPDADOMHFJPCEFNLKGHBGPBPKAA00&SELECT=S.sh\|&R=19&Process+Action=rss_feed)RSS Feed](http://ovidsp.uk.ovid.com/sp-3.13.0b/ovidweb.cgi?&S=BMAGPDADOMHFJPCEFNLKGHBGPBPKAA00&SELECT=S.sh%7c&R=19&Process+Action=rss_feed) |
|  | 20 | (((((pala* or panam*or papua new guinea or paragu* or per* or phillipen* or rwand* or samo* or sao tome) and principe) or Senegal* or Seychelles or sierra leon* or solomon islands or Somali* or south afric* or south suda* or sri lank* or st luc* or st vincen* or suda* or surina* or Swaziland or Syrian arab republic or tajikista* or tanzan* or thail* or timoe-leste or tog* or tong* or turkmensista* or tuval* or ugand* or uzbekist* or vanuat* or venezuel* or vietna* or west bank) and gaza) or yemen or zambi* or zimbabw*).kw,ti. | 6236 | Advanced | [[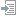](http://ovidsp.uk.ovid.com/sp-3.13.0b/ovidweb.cgi?&S=BMAGPDADOMHFJPCEFNLKGHBGPBPKAA00&SELECT=S.sh\|&R=20&Process+Action=display)Display](http://ovidsp.uk.ovid.com/sp-3.13.0b/ovidweb.cgi?&S=BMAGPDADOMHFJPCEFNLKGHBGPBPKAA00&SELECT=S.sh%7c&R=20&Process+Action=display)  [[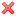](http://ovidsp.uk.ovid.com/sp-3.13.0b/ovidweb.cgi?&S=BMAGPDADOMHFJPCEFNLKGHBGPBPKAA00&SELECT=S.sh\|&R=20&Process+Action=delete)Delete](http://ovidsp.uk.ovid.com/sp-3.13.0b/ovidweb.cgi?&S=BMAGPDADOMHFJPCEFNLKGHBGPBPKAA00&SELECT=S.sh%7c&R=20&Process+Action=delete)  [[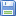](http://ovidsp.uk.ovid.com/sp-3.13.0b/ovidweb.cgi?&S=BMAGPDADOMHFJPCEFNLKGHBGPBPKAA00&SELECT=S.sh\|&R=20&Process+Action=save)Save](http://ovidsp.uk.ovid.com/sp-3.13.0b/ovidweb.cgi?&S=BMAGPDADOMHFJPCEFNLKGHBGPBPKAA00&SELECT=S.sh%7c&R=20&Process+Action=save)  [[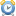](http://ovidsp.uk.ovid.com/sp-3.13.0b/ovidweb.cgi?&S=BMAGPDADOMHFJPCEFNLKGHBGPBPKAA00&SELECT=S.sh\|&R=20&Process+Action=autoalert)Auto-Alert](http://ovidsp.uk.ovid.com/sp-3.13.0b/ovidweb.cgi?&S=BMAGPDADOMHFJPCEFNLKGHBGPBPKAA00&SELECT=S.sh%7c&R=20&Process+Action=autoalert)  [[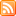](http://ovidsp.uk.ovid.com/sp-3.13.0b/ovidweb.cgi?&S=BMAGPDADOMHFJPCEFNLKGHBGPBPKAA00&SELECT=S.sh\|&R=20&Process+Action=rss_feed)RSS Feed](http://ovidsp.uk.ovid.com/sp-3.13.0b/ovidweb.cgi?&S=BMAGPDADOMHFJPCEFNLKGHBGPBPKAA00&SELECT=S.sh%7c&R=20&Process+Action=rss_feed) |
|  | 21 | (physical* or activit* or motor activit* or excerci* or movement or sedentary or inactivt* or walk* or sitt*).kw,ti. | 826019 | Advanced | [[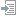](http://ovidsp.uk.ovid.com/sp-3.13.0b/ovidweb.cgi?&S=BMAGPDADOMHFJPCEFNLKGHBGPBPKAA00&SELECT=S.sh\|&R=21&Process+Action=display)Display](http://ovidsp.uk.ovid.com/sp-3.13.0b/ovidweb.cgi?&S=BMAGPDADOMHFJPCEFNLKGHBGPBPKAA00&SELECT=S.sh%7c&R=21&Process+Action=display)  [More ≫](http://ovidsp.uk.ovid.com/sp-3.13.0b/ovidweb.cgi) |
|  | 22 | 17 or 21 | 826019 | Advanced | [[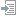](http://ovidsp.uk.ovid.com/sp-3.13.0b/ovidweb.cgi?&S=BMAGPDADOMHFJPCEFNLKGHBGPBPKAA00&SELECT=S.sh\|&R=22&Process+Action=display)Display](http://ovidsp.uk.ovid.com/sp-3.13.0b/ovidweb.cgi?&S=BMAGPDADOMHFJPCEFNLKGHBGPBPKAA00&SELECT=S.sh%7c&R=22&Process+Action=display)  [More ≫](http://ovidsp.uk.ovid.com/sp-3.13.0b/ovidweb.cgi) |
|  | 23 | 16 or 18 or 20 | 941504 | Advanced | [[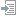](http://ovidsp.uk.ovid.com/sp-3.13.0b/ovidweb.cgi?&S=BMAGPDADOMHFJPCEFNLKGHBGPBPKAA00&SELECT=S.sh\|&R=23&Process+Action=display)Display](http://ovidsp.uk.ovid.com/sp-3.13.0b/ovidweb.cgi?&S=BMAGPDADOMHFJPCEFNLKGHBGPBPKAA00&SELECT=S.sh%7c&R=23&Process+Action=display)  [More ≫](http://ovidsp.uk.ovid.com/sp-3.13.0b/ovidweb.cgi) |
|  | 24 | 19 and 22 and 23 | 269 | Advanced | [[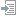](http://ovidsp.uk.ovid.com/sp-3.13.0b/ovidweb.cgi?&S=BMAGPDADOMHFJPCEFNLKGHBGPBPKAA00&SELECT=S.sh\|&R=24&Process+Action=display)Display](http://ovidsp.uk.ovid.com/sp-3.13.0b/ovidweb.cgi?&S=BMAGPDADOMHFJPCEFNLKGHBGPBPKAA00&SELECT=S.sh%7c&R=24&Process+Action=display)  [More ≫](http://ovidsp.uk.ovid.com/sp-3.13.0b/ovidweb.cgi) |
|  | 25 | 24 and "human" [Subjects] | 159 | Advanced | [[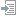](http://ovidsp.uk.ovid.com/sp-3.13.0b/ovidweb.cgi?&S=BMAGPDADOMHFJPCEFNLKGHBGPBPKAA00&SELECT=S.sh\|&R=25&Process+Action=display)Display](http://ovidsp.uk.ovid.com/sp-3.13.0b/ovidweb.cgi?&S=BMAGPDADOMHFJPCEFNLKGHBGPBPKAA00&SELECT=S.sh%7c&R=25&Process+Action=display) |

CINAHL – 1023 : SEPT 4^th^

| S22 | S13 AND S19 AND S20 | Limiters - Published Date: 19990101-20141231  Search modes - Boolean/Phrase | [View Results](javascript:__doPostBack('ctl00$ctl00$FindField$FindField$historyControl$HistoryRepeater$ctl00$linkResults','')) (1,032)  [View Details](javascript:showShDetails(%22ctl00_ctl00_FindField_FindField_historyControl_ctrlPopup%22,%20%22S22%22);)  [Edit](http://web.a.ebscohost.com/Legacy/Views/UserControls/EHOST/) |  |
| --- | --- | --- | --- | --- |
|  | S21 | S13 AND S19 AND S20 | Search modes - Boolean/Phrase | [View Results](javascript:__doPostBack('ctl00$ctl00$FindField$FindField$historyControl$HistoryRepeater$ctl01$linkResults','')) (1,081)  [View Details](javascript:showShDetails(%22ctl00_ctl00_FindField_FindField_historyControl_ctrlPopup%22,%20%22S21%22);)  [Edit](http://web.a.ebscohost.com/Legacy/Views/UserControls/EHOST/) |
|  | S20 | S16 OR S17 OR S18 | Search modes - Boolean/Phrase | [Rerun](javascript:__doPostBack('ctl00$ctl00$FindField$FindField$historyControl$HistoryRepeater$ctl02$linkResults',''))  [View Details](javascript:showShDetails(%22ctl00_ctl00_FindField_FindField_historyControl_ctrlPopup%22,%20%22S20%22);)  [Edit](http://web.a.ebscohost.com/Legacy/Views/UserControls/EHOST/) |
|  | S19 | Europe/ or *Russia/ or *Ukraine/ or *France/ or *Spain/ or *Sweden/ or *Norway/ or *Germany/ or *Finland/ or *Poland/ or *Italy/ or *Great Britain/ or *Romania/ or *"Republic of Belarus"/ or *Kazakhstan/ or *Greece/ or *Bulgaria/ or *Iceland/ or *Hungary/ or *Portugal/ or *Austria/ or *Czech Republic/ or *Serbia/ or *Ireland/ or *Latvia/ or *Bosnia-Herzegovina/ or *Croatia/ or *Lithuania/ or *Slovakia/ or *Estonia/ or *Denmark/ or *Netherlands/ or *Switzerland/ or *Moldova/ or *Belgium/ o [...](javascript:showHistoryTerm('ctl00_ctl00_FindField_FindField_historyControl_HistoryRepeater_ctl03_ellipsis',true)) | Search modes - Boolean/Phrase | [Rerun](javascript:__doPostBack('ctl00$ctl00$FindField$FindField$historyControl$HistoryRepeater$ctl03$linkResults',''))  [View Details](javascript:showShDetails(%22ctl00_ctl00_FindField_FindField_historyControl_ctrlPopup%22,%20%22S19%22);)  [Edit](http://web.a.ebscohost.com/Legacy/Views/UserControls/EHOST/) |
|  | S18 | ((((pala* or panam* or papua new guinea or paragu* or per* or phillipen* or rwand* or samo* or sao tome) and principe) or Senegal* or Seychelles or sierra leon* or solomon islands or Somali* or south afric* or south suda* or sri lank* or st luc* or st vincen* or suda* or surina* or Swaziland or Syrian arab republic or tajikista* or tanzan* or thail* or timoe-leste or tog* or tong* or turkmensista* or tuval* or ugand* or uzbekist* or vanuat* or venezuel* or vietna* or west bank) and gaza) or yeme [...](javascript:showHistoryTerm('ctl00_ctl00_FindField_FindField_historyControl_HistoryRepeater_ctl04_ellipsis',true)) | Search modes - Boolean/Phrase | [Rerun](javascript:__doPostBack('ctl00$ctl00$FindField$FindField$historyControl$HistoryRepeater$ctl04$linkResults',''))  [View Details](javascript:showShDetails(%22ctl00_ctl00_FindField_FindField_historyControl_ctrlPopup%22,%20%22S18%22);)  [Edit](http://web.a.ebscohost.com/Legacy/Views/UserControls/EHOST/) |
|  | S17 | ((((((*chinese*/ or *china/ or *irish traveller*/ or gyps*.mp. or *roma/ or *Asian/ or *Pakistan*/ or *banglades*/ or *turk*/ or *iran/ or *Iranian*/ or *irak*/ or *Maghreb*/ or *tunisi*/ or *morocco*/ or *liby*/ or *algeri*/ or *Surinam*/ or *latin American*/ or *cameroo*/ or *equado*/ or *eritre*/ or *chil*/ or *estoni*/ or *latvi*/ or *lithuani*/ or *goergi*/ or *armeni*/ or *azerbaija*/ or *kazakhsta*/ or *ukrain*/ or *belaru*/ or *moldov*/ or *croati*/ or *Czech republic*/ or *pol* [...](javascript:showHistoryTerm('ctl00_ctl00_FindField_FindField_historyControl_HistoryRepeater_ctl05_ellipsis',true)) | Search modes - Boolean/Phrase | [Rerun](javascript:__doPostBack('ctl00$ctl00$FindField$FindField$historyControl$HistoryRepeater$ctl05$linkResults',''))  [View Details](javascript:showShDetails(%22ctl00_ctl00_FindField_FindField_historyControl_ctrlPopup%22,%20%22S17%22);)  [Edit](http://web.a.ebscohost.com/Legacy/Views/UserControls/EHOST/) |
|  | S16 | *"Emigrants and Immigrants"/ or *cultural diversity/ or *Minority Groups/ or *"Transients and Migrants"/ or *Ethnic Groups/ or multiculturalism*.mp. or *ethnic minorit*/ or BME.mp. or black minorit*.mp. or ethnic*.mp. or asylum seeker*.mp. or refugee*.mp. or african caribbean*.mp. or *West Indies/ or *Afro-caribbean*/ or *Non-white/ or *Coloured population/ or *Black*/ or *Afric*/ or *Indi*/ or *Caucasian*/ or *Caribbean*/ or *Arab*/ or *Black Afric*/ or *South Asia*/ or *Trinidad/) an [...](javascript:showHistoryTerm('ctl00_ctl00_FindField_FindField_historyControl_HistoryRepeater_ctl06_ellipsis',true)) | Search modes - Boolean/Phrase | [Rerun](javascript:__doPostBack('ctl00$ctl00$FindField$FindField$historyControl$HistoryRepeater$ctl06$linkResults',''))  [View Details](javascript:showShDetails(%22ctl00_ctl00_FindField_FindField_historyControl_ctrlPopup%22,%20%22S16%22);)  [Edit](http://web.a.ebscohost.com/Legacy/Views/UserControls/EHOST/) |
|  | S15 | S9 AND S13 AND S14 | Search modes - Boolean/Phrase | [Rerun](javascript:__doPostBack('ctl00$ctl00$FindField$FindField$historyControl$HistoryRepeater$ctl07$linkResults',''))  [View Details](javascript:showShDetails(%22ctl00_ctl00_FindField_FindField_historyControl_ctrlPopup%22,%20%22S15%22);)  [Edit](http://web.a.ebscohost.com/Legacy/Views/UserControls/EHOST/) |
|  | S14 | S7 OR S8 OR S10 | Search modes - Boolean/Phrase | [Rerun](javascript:__doPostBack('ctl00$ctl00$FindField$FindField$historyControl$HistoryRepeater$ctl08$linkResults',''))  [View Details](javascript:showShDetails(%22ctl00_ctl00_FindField_FindField_historyControl_ctrlPopup%22,%20%22S14%22);)  [Edit](http://web.a.ebscohost.com/Legacy/Views/UserControls/EHOST/) |
|  | S13 | S11 OR S12 |  |  |
