# Supplementary material for: Systematic mapping review of the factors influencing physical activity and sedentary behaviour in ethnic minority groups in Europe: a DEDIPAC study
Source: Int J Behav Nutr Phys Act. 2017 Jul 24;14:99. doi: 10.1186/s12966-017-0554-3 (PMC5525226; doi:10.1186/s12966-017-0554-3)
Supplement: Supplementary file 2 — Characteristics of quantitative and qualitative studies. (DOCX 17 kb) [file 12966_2017_554_MOESM2_ESM.docx]

Additional file 2: Characteristics of quantitative and qualitative studies

| **Characteristic** | | **Quantitative** | **Qualitative** | **Total** |
| --- | --- | --- | --- | --- |
| **Gender** | Men and women | 32 | 13 | 45 |
|  | Women | 5 | 9 | 14 |
|  | Men | 4 | 0 | 4 |
|  | Total | 41 | 22 | 63 |
|  |  |  |  |  |
| **Age/life stage** | Children^1^ | 9 | 4 | 13 |
|  | Adolescents | 4 | 2 | 6 |
|  | Adults | 24 | 11 | 35 |
|  | Young older adults^2^ | 1 | 3 | 4 |
|  | Older adults | 1 | 1 | 2 |
|  | Population | 2 | 1 | 3 |
|  | Total | 41 | 22 | 63 |
|  |  |  |  |  |
| **Country of research** | Western Europe | 17 | 16 | 33 |
|  | Nordic countries | 17 | 2 | 19 |
|  | Comparative | 3 | 4 | 7 |
|  | European countries/various | 3 | 0 | 3 |
|  | Middle Europe | 1 | 0 | 1 |
|  | Eastern Europe | 0 | 0 | 0 |
|  | Southern Europe | 0 | 0 | 0 |
|  | Total | 41 | 22 | 63 |
|  |  |  |  |  |
| **Study population by country of origin, region, ethnicity and/or religion** | South Asia | 19 | 14 | 33 |
|  | North Europe | 13 | 2 | 15 |
|  | Middle East | 10 | 2 | 12 |
|  | General population | 8 | 1 | 9 |
|  | Other origin (not specified) | 8 | 0 | 8 |
|  | (Black) African (not specified) | 6 | 3 | 9 |
|  | (White) European (not specified) | 4 | 0 | 4 |
|  | North Africa | 3 | 1 | 4 |
|  | Muslim | 0 | 4 | 4 |
|  | West Europe | 3 | 0 | 3 |
|  | South America | 2 | 1 | 3 |
|  | Caucasian/white | 2 | 1 | 3 |
|  | Southern Europe | 1 | 1 | 2 |
|  | Sami | 2 | 0 | 2 |
|  | Asian (not specified) | 0 | 1 | 1 |
|  | East Europe | 1 | 0 | 1 |
|  | East Asia | 1 | 0 | 1 |
|  | West Africa | 0 | 1 | 1 |
|  | East Africa | 1 | 0 | 1 |
|  | Asian Muslim | 0 | 1 | 1 |
|  | South East Asia | 1 | 0 | 1 |
|  | Total | 85 | 33 | 118 |

^1^ Includes articles with both children and adolescents

^2^ Age 60-70 years
